# Meta-analysis and public policy: Reconciling the evidence on deworming

**DOI:** 10.1073/pnas.2308733121

**Published:** 2024-06-10

**Authors:** Kevin Croke, Joan Hamory, Eric Hsu, Michael Kremer, Ricardo Maertens, Edward Miguel, Witold Więcek

**Affiliations:** ^a^Department of Global Health and Population, Harvard T.H. Chan School of Public Health, Boston, MA 02115; ^b^Department of Economics, University of Oklahoma, Norman, OK 73019; ^c^Department of Economics, University of California, Berkeley, CA 94720; ^d^Department of Economics, University of Chicago, Chicago, IL 60637; ^e^National Bureau of Economic Research, Cambridge, MA 02138; ^f^Department of Economics, Harvard University, Cambridge, MA 02138

**Keywords:** meta-analysis, cost-effectiveness, deworming, nutrition

## Abstract

The WHO recommends mass drug administration (MDA) for intestinal worm infections in areas with over 20% infection prevalence. Recent Cochrane meta-analyses endorse treatment of infected individuals but recommend against MDA. We conducted a theory-agnostic random-effects meta-analysis of the effect of multiple-dose MDA and a cost-effectiveness analysis. We estimate significant effects of MDA on child weight (0.15 kg, 95% CI: 0.07, 0.24; *P* < 0.001), mid-upper arm circumference (0.20 cm, 95% CI: 0.03, 0.37; *P* = 0.02), and height (0.09 cm, 95% CI: 0.01, 0.16; *P* = 0.02) when prevalence is over 20% but not on Hb (0.06 g/dL, 95% CI: −0.01, 0.14; *P* = 0.1). These results suggest that MDA is a cost-effective intervention, particularly in the settings where it is recommended by the WHO.

## Significance

Soil-transmitted helminths infect 1 in 4 people in endemic countries. Worm infections adversely affect child health and development. Because testing is much more expensive than treatment, mass drug administration (MDA) has been proposed as a cost-effective approach to deworming. However, there has been a debate over evidence on the health benefits of MDA approaches. In this meta-analysis, we reaffirm significant positive health impacts of MDA and show that it is cost-effective.

Soil-transmitted helminths (STH; including hookworm, whipworm, and roundworm) infect 1 in 4 people in endemic countries (1). STH are spread via eggs deposited in the local environment through feces. School-aged children are especially vulnerable to infections and play an important role in local transmission (2). Worm infections affect child health and nutrition through impaired nutritional intake, reduced nutrient absorption, intestinal damage, dysentery, blood loss, and combinations of these pathways, depending on the worm species (3).

The most common drugs used to treat STH, albendazole and mebendazole (3), are extremely well tolerated by infected and noninfected individuals (4). Side effects are very infrequent (about 1%), not severe (e.g., nausea, rashes), mainly related to the elimination of heavy worm loads, and typically disappear within 48 h (4–6).

There is agreement that children known to be infected should be treated; indeed, this is the standard of medical care (7–9). Furthermore, the World Health Organization (WHO) has long recommended mass drug administration (MDA) for intestinal worms among children in areas with more than 20% infection prevalence (1 annual dose) or more than 50% prevalence (2 annual doses).^*^ Standard testing methods require skilled staff and laboratory facilities, and sensitivity is 52 to 91% (10, 11). Therefore, many infections would go undetected even with screening. Moreover, the cost of screening for worm infections is 4 to 10 times that of treatment (12).

After the WHO recommendation, a social science literature emerged measuring the long-term educational and economic impacts of mass deworming, suggesting that the benefits of MDA far exceed the costs (13–16). Three studies in moderate to high prevalence settings—in Kenya and the (historical) southern United States—find substantial long-run impacts of deworming on educational outcomes (14, 15, 17). Several of these studies also report economic outcomes and find positive effects; we give more detail on these results in *SI Appendix*, section G.

Recent meta-analyses have cast doubt on the WHO’s recommendation. Taylor-Robinson et al. (12) estimate that single-dose treatment for children known to be infected leads to statistically significant gains across various nutritional outcomes and express support for treating these children (p. 30). However, they argue that there is “substantial evidence” that MDA has no impact on child outcomes (p. 3) and recommend against its implementation (p. 30). This creates an apparent paradox: If infected individuals benefit, then one would expect a smaller—but still positive—average effect of MDA in endemic populations. The paradox remains in the 2019 update of the review, where the authors argue that it is “obvious” (p. 29) that children known to be infected with worms should receive treatment but reaffirm their recommendation against mass treatment in endemic populations (18).

In this paper, we conduct a meta-analysis of trials of multiple-dose MDA with outcomes measured at longest follow-up. We limit the analysis to trials that report effects on children’s weight, mid-upper arm circumference (MUAC), height, or hemoglobin (Hb). A well-known limitation of meta-analyses, particularly in health research, is that they are commonly underpowered to detect the treatment effects (19). By following the *Cochrane Handbook for Systematic Reviews of Interventions*, we strengthen the analysis in several ways (20). First, we include studies identified by Taylor-Robinson et al. (12, 18) but excluded from their meta-analysis (for instance, because SEs were not directly reported in the study, even though they could be calculated from other reported statistics). Second, we extract point estimates and SEs of the impact of MDA using the most precise estimators available (e.g., ANCOVA, difference-in-differences). Consequently, the statistical analysis is better powered to detect nutritional gains from deworming than previous meta-analyses (12, 18, 21). Using the meta-analytic result, we then compare the estimated health gains per dollar spent from MDA in settings with over 20% worm prevalence to school feeding, another widely implemented intervention that targets similar outcomes in similar populations (22, 23).^†^

## 1. Results

As a result of this data extraction step, we include five studies not present in Taylor-Robinson et al. (18). Moreover, we extract different estimates from another eight studies. We discuss these differences further at the end of this section and *SI Appendix*, section A.

Table 1 presents data on all outcomes and prevalence values used for statistical analyses. With regard to child nutrition effects of MDA, we include 27 estimates (22 trials) for weight, 7 estimates (6 trials) for MUAC, 22 estimates (17 trials) for height, and 13 estimates (9 trials) for hemoglobin.^‡^ Dispersion is large across mean treatment effects of all outcomes (weight: ranging from −0.5 to 0.9 kg; height: from −1.2 to 1.4 cm; MUAC: from −0.4 to 0.8 cm; Hb: from −0.1 to 0.3 g/dL). Prevalence among MDA studies ranges from 3% to 95%: 6 MDA studies have less than 20% prevalence, 6 studies have between 20% and 50%, and 19 studies have more than 50%. The sample also includes six test-and-treat trials.

**Table:** Table 1. Summary of treatment effects and prevalence of worms in included studies

	Weight (kg)		Height (cm)		Mid-Upper arm circumference (cm)		Hemoglobin (g/dL)		
Study	N	Treatment effects (SE)	N	Treatment effects (SE)	N	Treatment effects (SE)	N	Treatment effects (SE)	Worm prevalence (%)
Panel A: MDA trials									
Alderman 2006	48^†^	0.154 (0.089)							76%
Awasthi 1995/2008	50^†^	0.980 (0.148)	50^†^	1.190 (1.204)					8%
Awasthi 2000	1,045	−0.050 (0.076)	1,045	−0.410 (0.314)			1,045	0.000 (0.041)	12%
Awasthi 2001	124^†^	0.170 (0.065)	124^†^	0.400 (0.310)					9%
Carmona-Fonseca 2015a	603	0.201 (0.136)	601	−0.067 (0.193)			586	0.129 (0.091)	45%
Carmona-Fonseca 2015b	658	0.062 (0.118)	657	−0.067 (0.193)			624	0.007 (0.082)	45%
Donnen 1998	198	−0.450 (0.167)	198	−1.190 (0.552)	198	−0.350 (0.154)			10%
Dossa 2001a	65	0.000 (0.265)	65	0.500 (0.637)	65	0.000 (0.215)	70	0.300 (0.299)	58%
Dossa 2001b	64	0.000 (0.139)	64	0.000 (0.317)	64	0.100 (0.188)	68	0.200 (0.329)	58%
Gateff 1972	280	0.347 (0.131)							76%
Gupta 1982a	78	0.027 (0.175)	78	−0.095 (0.444)					62%
Gupta 1982b	81	0.130 (0.148)	81	−0.029 (0.474)					59%
Hall 2006	80^†^	0.054 (0.058)	80^†^	0.089 (0.082)	80^†^	0.794 (0.314)			84%
Joseph 2015	777	0.040 (0.049)	777	0.040 (0.127)					11%
Kirwan 2010							320	0.170 (0.121)	46%
Kruger 1996a	74	−0.376 (0.248)	74	0.086 (0.218)			74	−0.020 (0.154)	38%
Kruger 1996b	104	0.393 (0.186)	104	0.209 (0.208)			104	0.270 (0.129)	38%
Le Huong 2007a							161	−0.080 (0.136)	73%
Le Huong 2007b							165	−0.029 (0.129)	73%
Liu 2017	112^†^	0.030 (0.127)	112^†^	0.080 (0.352)			112^†^	−0.043 (0.108)	31%
Miguel 2004	50^†^	−0.660 (0.300)		1.546 (0.535)					77%
Ndibazza 2012	1,228	0.010 (0.091)	1,210	−0.230 (0.285)			1,109	−0.070 (0.095)	3%
Ostwald 1984	87	0.700 (0.449)	86	0.300 (0.270)			70	0.300 (0.277)	92%
Rousham 1994		−0.090 (0.043)		0.080 (0.063)	13^*†^	0.100 (0.058)			71%
Stephenson 1993	188	0.900 (0.184)	188	−0.100 (0.163)	188	0.400 (0.065)			88%
Stoltzfus 1997a	12^†^	0.234 (0.098)	12^†^	0.218 (0.086)					95%
Stoltzfus 1997b	12^†^	0.110 (0.139)	12^†^	−0.000 (0.098)					95%
Sur 2005	682	0.290 (0.090)							53%
Watkins 1996	226	0.130 (0.106)	227	0.060 (0.098)	207	0.080 (0.070)			91%
Willett 1979	273	0.160 (0.085)							53%
Wiria 2013	954^†^	0.188 (0.394)	954^†^	1.348 (0.535)					76%
Freij 1979a		0.200 (1.470)				−0.300 (0.713)			49%
Freij 1979b						0.100 (0.347)			49%
Sarkar 2002		0.380 (0.150)		0.100 (0.261)					79%
Stephenson 1989		1.300 (0.134)		0.600 (0.134)		0.500 (0.078)			97%
Tee 2013				−0.100 (0.404)					31%
Yap 2014		0.300 (0.179)		0.200 (0.128)				−0.400 (0.434)	93%

Notes: For each study, the worm prevalence is defined as the maximum of prevalences over all worms reported in the study. Values in column N represent the number of individuals, with the exception of values marked with a (^†^), which indicate the number of clusters.

### 1.1. Estimation of the Mean Effect of MDA.

When estimating the mean effect of MDA on child nutrition indicators, we report results both in the set of trials that take place in settings where the WHO recommends deworming (i.e., those where the baseline prevalence of hookworm, whipworm, or roundworm is over the 20% threshold for annual MDA) and in the full sample. When examining the evidence on deworming infected children, we pool evidence from MDA trials with that of deworming trials of children who were screened for infection (“test-and-treat” trials).

**Fig. 1.**
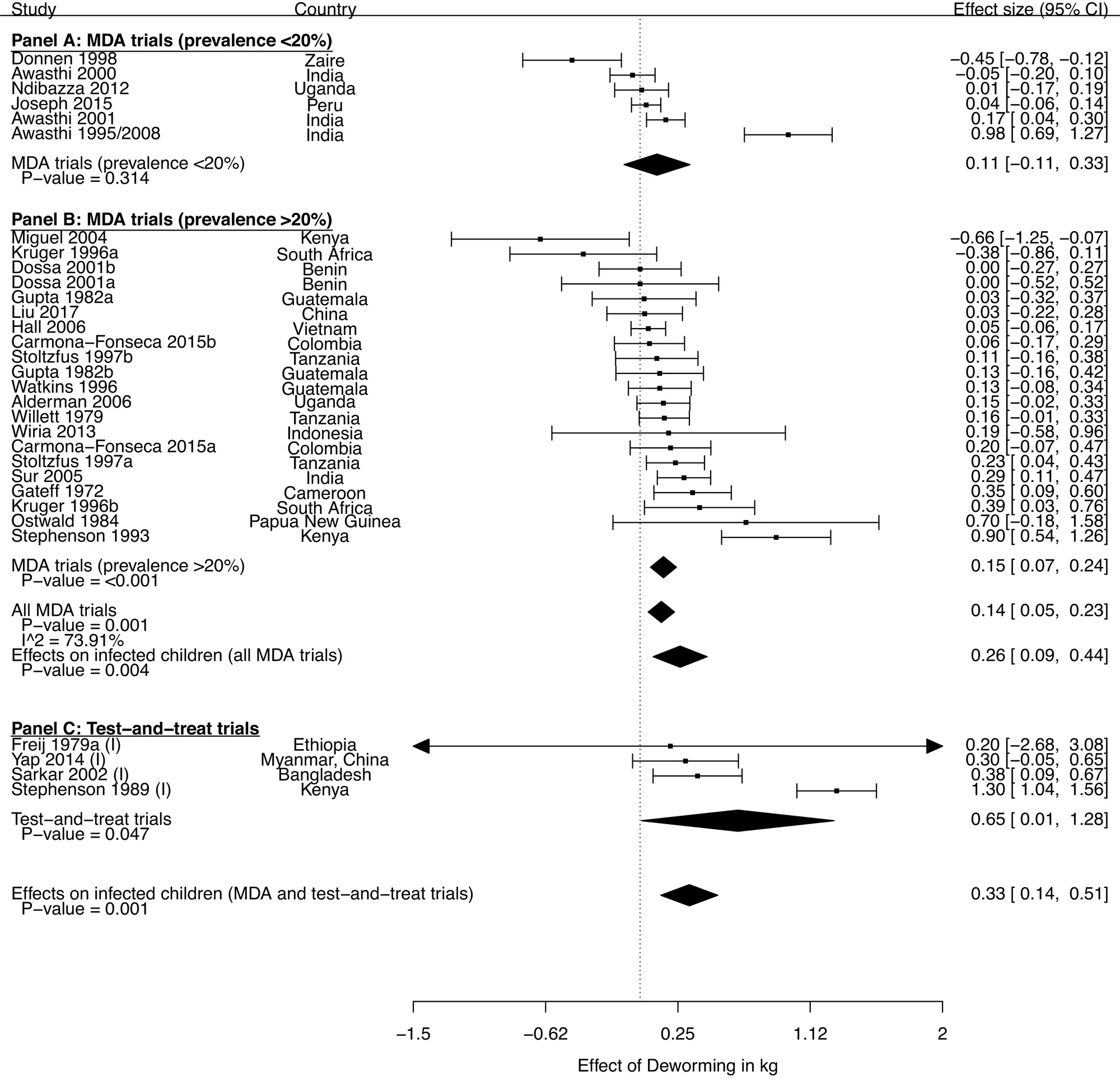
Forest plot of the effect of deworming on weight (kg). Notes: Panel *A* shows results from MDA trials conducted in settings where average prevalence is below 20%. Panel *B* shows results from MDA trials conducted in settings where average prevalence is above 20%. Panel *C* shows results from test-and-treat trials. We show estimated mean effects for each subgroup, and we also estimate mean effects for all MDA trials (including trials conducted in settings above and below 20% prevalence). In addition, we estimate mean effects for infected children using all MDA and test-and-treat trials. To estimate the mean effect on infected children, point estimates and SEs from MDA trials were divided by infection prevalence prior to applying a random effects model. All mean effects are estimated using a random effects model. Arrows indicate that the CI is larger than what is displayed on the graph.

**Fig. 2.**
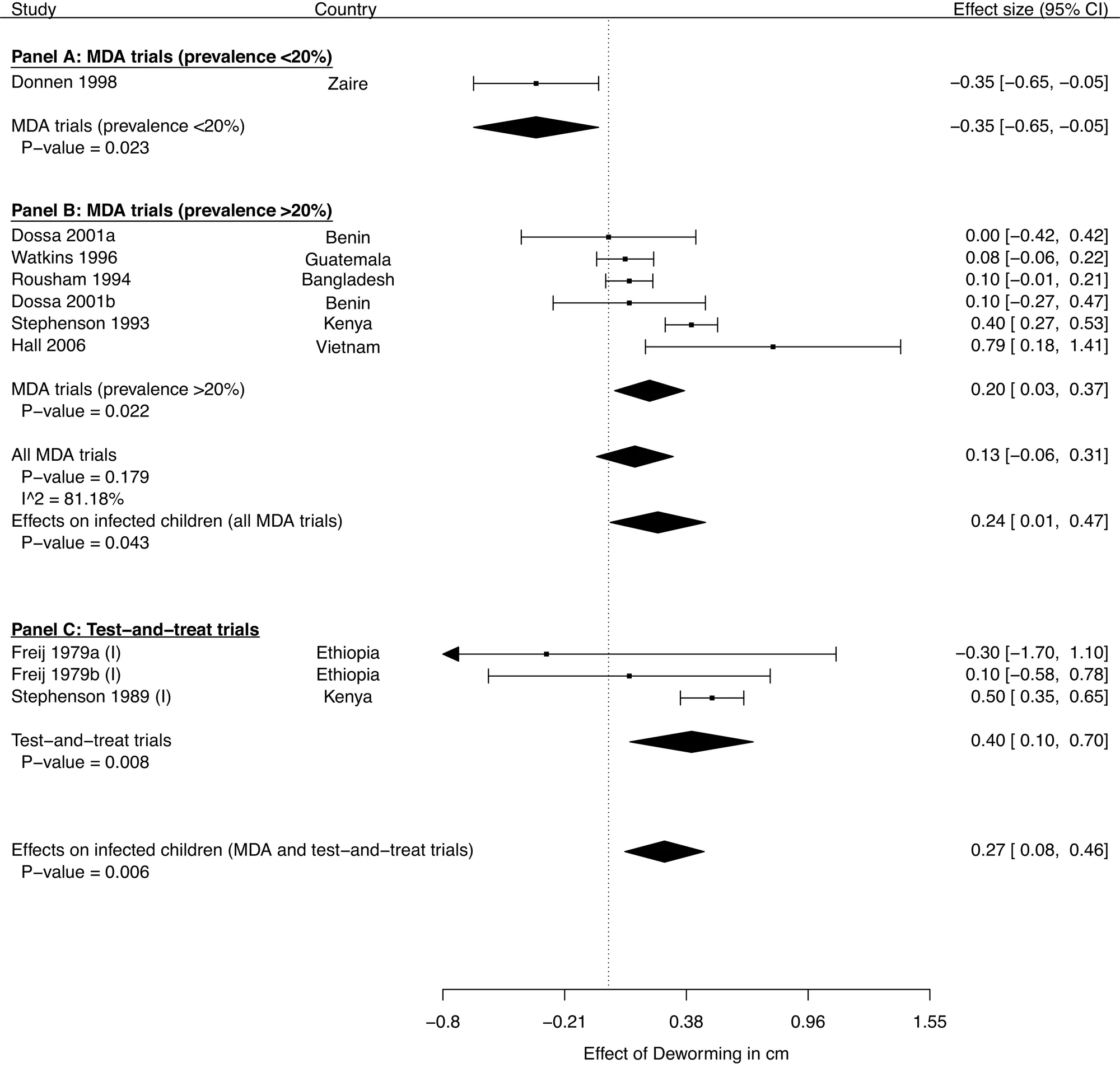
Forest plot of the effect of deworming on MUAC (cm). Notes: Panel *A* shows results from MDA trials conducted in settings where average prevalence is below 20%. Panel *B* shows results from MDA trials conducted in settings where average prevalence is above 20%. Panel *C* shows results from test-and-treat trials. We show estimated mean effects for each subgroup, and we also estimate mean effects for all MDA trials (including trials conducted in settings above and below 20% prevalence). In addition, we estimate mean effects for infected children using all MDA and test-and-treat trials. To estimate the mean effect on infected children, point estimates and SEs from MDA trials were divided by infection prevalence prior to applying a random effects model. All mean effects are estimated using a random effects model. Arrows indicate that the CI is larger than what is displayed on the graph.

**Fig. 3.**
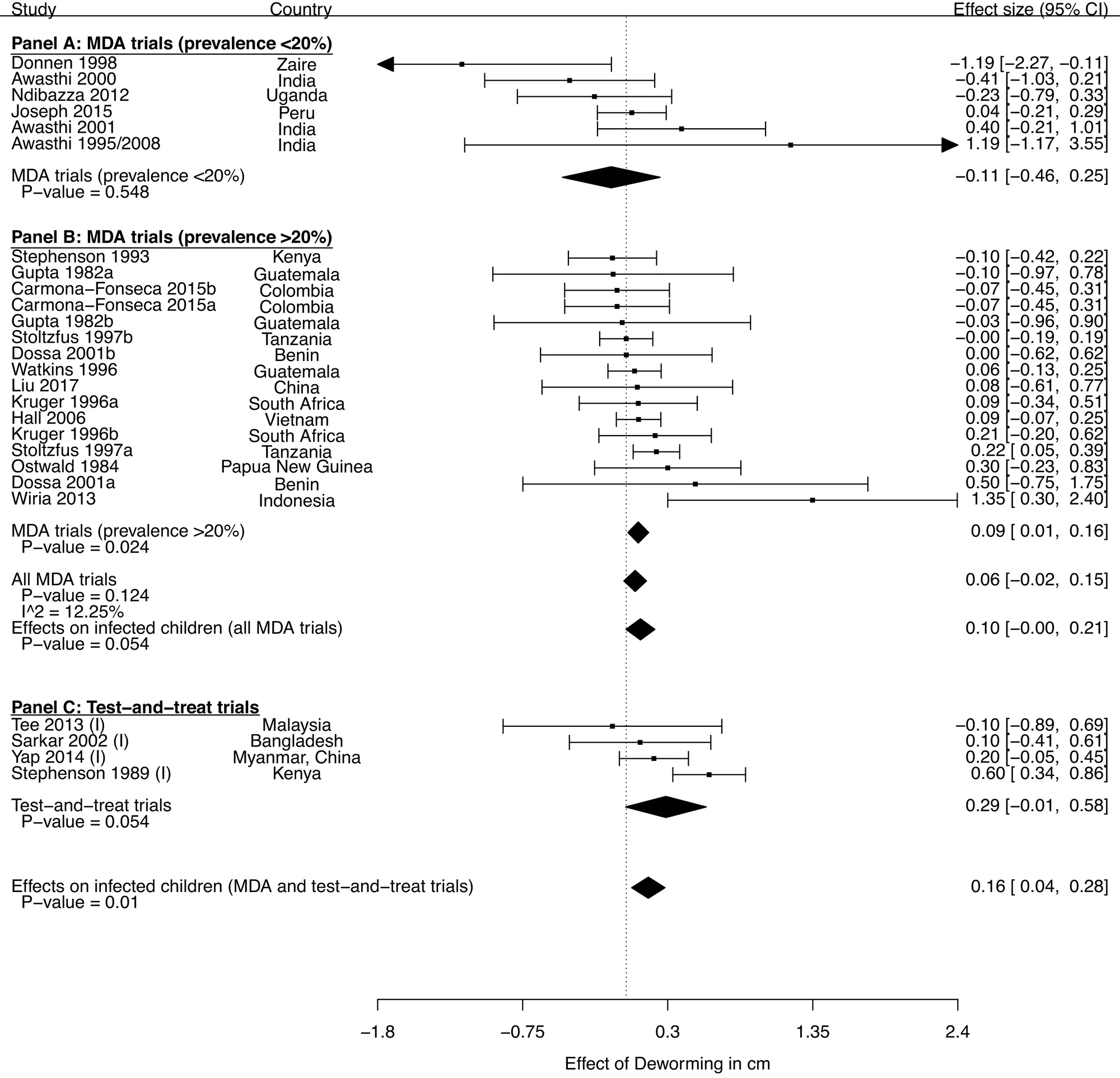
Forest plot of the effect of deworming on height (cm). Notes: Panel *A* shows results from MDA trials conducted in settings where average prevalence is below 20%. Panel *B* shows results from MDA trials conducted in settings where average prevalence is above 20%. Panel *C* shows results from test-and-treat trials. We show estimated mean effects for each subgroup, and we also estimate mean effects for all MDA trials (including trials conducted in settings above and below 20% prevalence). In addition, we estimate mean effects for infected children using all MDA and test-and-treat trials. To estimate the mean effect on infected children, point estimates and SEs from MDA trials were divided by infection prevalence prior to applying a random effects model. All mean effects are estimated using a random effects model. Arrows indicate that the CI is larger than what is displayed on the graph.

**Fig. 4.**
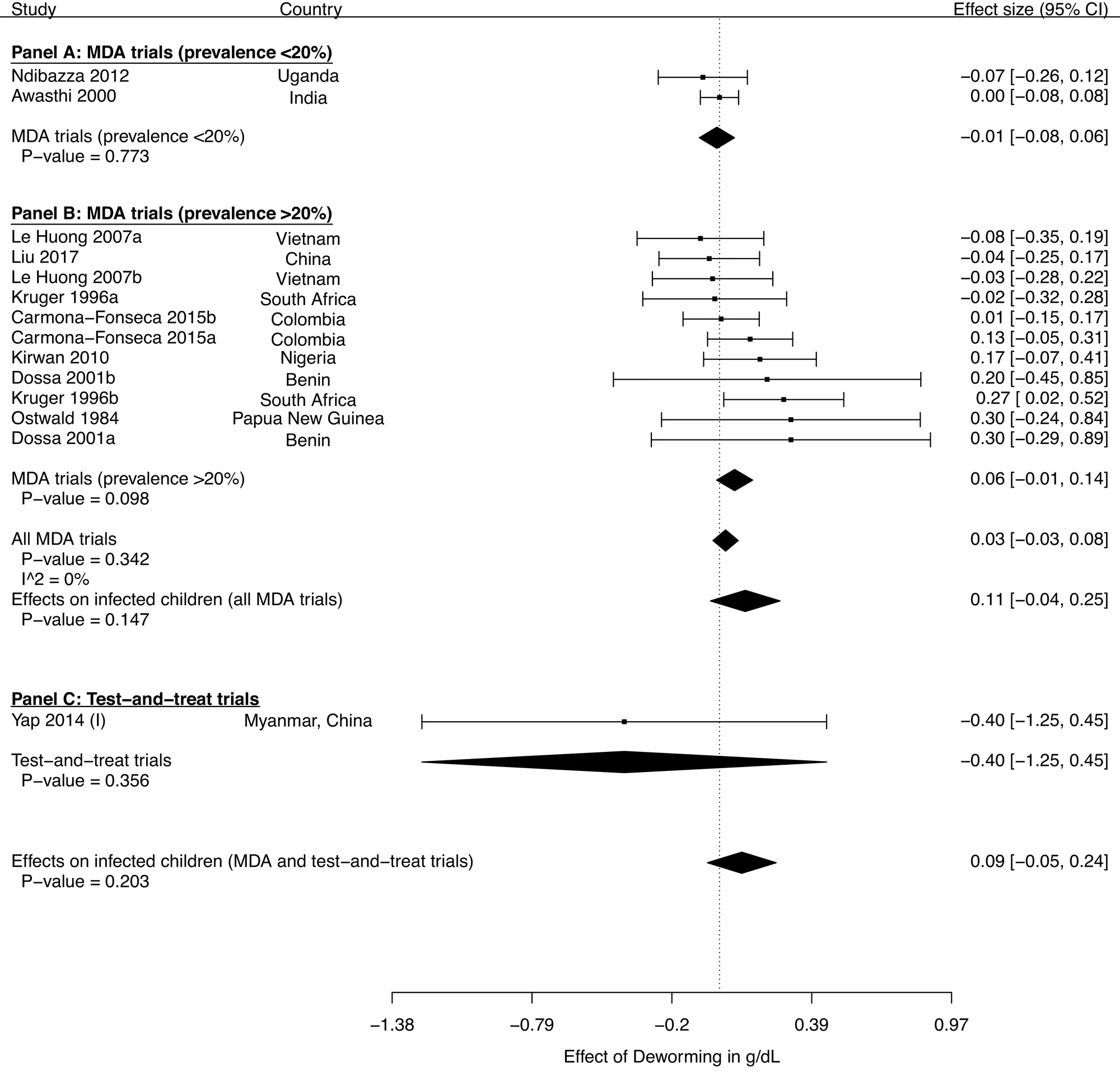
Forest plot of the effect of deworming on hemoglobin (g/dL). Notes: Panel *A* shows results from MDA trials conducted in settings where average prevalence is below 20%. Panel *B* shows results from MDA trials conducted in settings where average prevalence is above 20%. Panel *C* shows results from test-and-treat trials. We show estimated mean effects for each subgroup, and we also estimate mean effects for all MDA trials (including trials conducted in settings above and below 20% prevalence). In addition, we estimate mean effects for infected children using all MDA and test-and-treat trials. To estimate the mean effect on infected children, point estimates and SEs from MDA trials were divided by infection prevalence prior to applying a random effects model. All mean effects are estimated using a random effects model. Arrows indicate that the CI is larger than what is displayed on the graph.

Table 2 presents results for all meta-analysis models. Figs. 1–4 show forest plots of the effect of deworming from MDA and test-and-treat trials on all outcomes. In the RE models using the full sample of MDA studies, we find large values for the heterogeneity statistic I^2^ (the portion of variation explained by variation in true means) for models of weight (I^2^ = 74%) and MUAC (I^2^ = 81%) but not for height (I^2^ = 12%) and hemoglobin (I^2^ = 1%). Since low estimated heterogeneity may also be due to large sampling variation and small sample sizes, below we focus on presenting the RE results.

**Table 2.** Random-effects and fixed-effect estimates

	Weight (kg)		MUAC (cm)		Height (cm)		Hb (g/dL)	
	(1)	(2)	(3)	(4)	(5)	(6)	(7)	(8)
Estimation method	RE	FE	RE	FE	RE	FE	RE	FE
Panel A: Full sample of MDA trials								
Point estimate	0.141	0.117	0.127	0.164	0.064	0.071	0.026	0.026
SE	0.044	0.020	0.095	0.034	0.042	0.036	0.027	0.027
*P*-value^*^	0.001	0.000	0.179	0.000	0.124	0.048	0.342	0.342
	[<0.001]	[<0.001]	[0.09]	[<0.001]	[0.062]	[0.024]	[0.171]	[0.171]
	N = 27		N = 7		N = 22		N = 13	
Panel B: MDA trials with <20% prevalence								
Point estimate	0.112	0.076	−0.350	−0.350	−0.109	−0.035	−0.011	−0.011
SE	0.111	0.031	0.154	0.154	0.181	0.101	0.038	0.038
*P*-value*	0.314	0.015	0.023	0.023	0.548	0.729	0.773	0.773
	[0.157]	[0.008]	[0.988]	[0.988]	[0.726]	[0.636]	[0.614]	[0.614]
	N = 6		N = 1		N = 6		N = 2	
Panel C: MDA trials with ≥20% prevalence								
Point estimate	0.154	0.147	0.198	0.191	0.087	0.087	0.064	0.064
SE	0.044	0.027	0.086	0.035	0.039	0.039	0.038	0.038
*P*-value^*^	0.000	0.000	0.022	0.000	0.024	0.024	0.098	0.098
	[<0.001]	[<0.001]	[0.011]	[<0.001]	[0.012]	[0.012]	[0.049]	[0.049]
	N = 21		N = 6		N = 16		N = 11	
Panel D: MDA trials with ≥50% prevalence								
Point estimate	0.173	0.157	0.198	0.191	0.095	0.096	0.020	0.020
SE	0.051	0.029	0.086	0.035	0.048	0.042	0.082	0.082
*P*-value^*^	0.001	0.000	0.022	0.000	0.049	0.022	0.804	0.804
	[<0.001]	[<0.001]	[0.011]	[<0.001]	[0.025]	[0.011]	[0.402]	[0.402]
	N = 16		N = 6		N = 11		N = 5	
Panel E: Test-and-treat trials								
Point estimate	0.646	0.748	0.401	0.472	0.287	0.337	−0.400	−0.400
SE	0.325	0.087	0.152	0.076	0.149	0.085	0.434	0.434
*P*-value^*^	0.047	0.000	0.008	0.000	0.054	0.000	0.356	0.356
	[0.023]	[<0.001]	[0.004]	[<0.001]	[0.027]	[<0.001]	[0.822]	[0.822]
	N = 4		N = 3		N = 4		N = 1	
Panel F: Pooling all MDA and test-and-treat trials								
Point estimate	0.194	0.150	0.174	0.217	0.102	0.111	0.024	0.024
SE	0.053	0.020	0.089	0.031	0.048	0.033	0.027	0.027
*P*-value^*^	0.000	0.000	0.051	0.000	0.035	0.001	0.373	0.373
	[<0.001]	[<0.001]	[0.025]	[<0.001]	[0.018]	[<0.001]	[0.187]	[0.187]
	N = 31		N = 10		N = 26		N = 14	

^*^The *P*-value of the one-tailed test of the hypothesis of no effect against the alternative of a positive effect is presented in square brackets. The random-effects and fixed-effect estimates for the height and hemoglobin effects, in settings with over 20% worm prevalence (Panel B), are nearly identical (identical up to three decimal points) given that the estimated between-trial variances are small: 0.0043 for height and 0.0001 for hemoglobin. In the case of hemoglobin, the two estimates are also nearly identical in the other settings.

Notes: Estimation method is random-effects (RE) in odd numbered columns and fixed-effect (FE) in even numbered columns.

In the full sample of MDA trials (Table 2, Panel A), we find a positive and significant (at the conventional 5% significance level) effect on weight gain, where the mean effect is 0.14 kg (95% CI: 0.05, 0.23; *P* = 0.001). We find positive but insignificant increases on MUAC, 0.13 cm (95% CI: −0.06, 0.31; *P* = 0.18), on height: 0.06 cm (95% CI: −0.02, 0.15; *P* = 0.12), and on hemoglobin: 0.03 g/dL (95% CI: −0.03, 0.08; *P* = 0.34).

In MDA trials with over 20% prevalence (Table 2, panel C), where the WHO currently recommends MDA, the estimated mean treatment effects are somewhat larger and significant (with exception of hemoglobin): for weight, 0.15 kg (95% CI: 0.07, 0.24; *P* < 0.001), for MUAC, 0.20 cm (95% CI: 0.03, 0.37; *P* = 0.02), for height, 0.09 cm (95% CI: 0.01, 0.16; *P* = 0.02), for hemoglobin, 0.06 g/dL (95% CI: −0.01, 0.14; *P* = 0.1). In *SI Appendix*, section D, we show that the weight effect remains significant after dropping any one study estimate and after dropping any pair of estimates (and in most such comparisons for MUAC and height).

Estimates in test-and-treat trials (Table 2, Panel E) are positive, significant, and over twice as large as those of MDA trials for weight, MUAC, and height. We also report treatment effects in areas with prevalence greater than 50% (Table 2, Panel D) and below 20% (Table 2, Panel B), as well as pooling MDA and test-and-treat trials (panel F). As expected, the effects are typically larger for settings with higher prevalence.

We report the full results of an additional Bayesian meta-analysis using half-normal and skew-normal distributions of effects in *SI Appendix*, section E, where we find large increases in effect size for weight gain (from 0.14 to 0.21 kg) and MUAC (from 0.13 to 0.23 cm).

To contextualize the estimated effects of MDA on weight and height, we compare them to the largest and smallest difference in annual reference weight and height gains by gender (according to WHO growth charts), from birth to age 5, between children at the 15th and 50th percentiles of the respective distribution. The largest difference in annual weight gain between the 15th and 50th percentile is 0.6 kg (for boys and girls from birth to age 1); the smallest difference is 0.2 kg (for boys from age 2 to 3). The estimated MDA effect of 0.15 kg is 26% of the largest annual weight gain gap and 77% of the smallest gap. For height, the largest and smallest 15th to 50th percentile annual growth differences are 0.8 cm and 0.4 cm; the estimated effect of 0.06 cm is 7.5% of the larger (0.8) cm gap and 15% of the smaller (0.4) cm gap.

Random-effects meta-analyses of the implied effect on infected children (where effects are divided by prevalence) are presented in *SI Appendix*, Table S4. We find that, on average, among infected MDA increased child weight by 0.27 kg (*P*-value = 0.004), MUAC by 0.24 cm (*P*-value = 0.043), height by 0.10 cm (*P*-value = 0.054), and hemoglobin by 0.11 g/dL (*P*-value = 0.147). These effects are 60 to 90% larger than the effect in the full sample of MDA trials, without adjustment for prevalence (Table 2), except for hemoglobin, where the implied effect is over four times higher.

However, these estimates are still lower (again with exception of hemoglobin, where there is only one trial) than in test-and-treat trials (Table 2, panel E), where we estimate that deworming increases weight by 0.65 kg (*P*-value = 0.05), MUAC by 0.40 cm (*P*-value = 0.008), and height by 0.29 cm (*P*-value = 0.054).^§^^,^^¶^ We present results of standard publication bias analyses and standard funnel plots in *SI Appendix*, section F. None of the tests suggest the presence of publication bias. We also use a method from Andrews and Kasy (24) to obtain bias-adjusted estimates of effect and generally find they do not differ from our main estimates (24).

### 1.2. Cost-Effectiveness Analysis.

We estimate that the gains in child nutrition outcomes per $1,000 spent in deworming treatment are several times larger than those estimated for school and preschool feeding (Table 3). Deworming estimates are based on the random-effects estimates of the effect of MDA on nutrition outcomes, adjusting for the average number of deworming doses, and assuming a cost of $0.68 per person treated for two doses per year.^#^ In settings with over 20% worm prevalence, we find that a $1,000 investment in MDA results in nutritional gains of 144.6 kg of weight, 166.5 cm of MUAC, 80.0 cm of height, and 76.3 g/dL of hemoglobin (column 3). With exception of hemoglobin, gains are only slightly smaller when also including low prevalence settings (*SI Appendix*, Table S5).

**Table 3.** Cost-effectiveness analysis

	Deworming MDA (≥20% prevalence settings)			Deworming MDA (≥50% prevalence settings)			School feeding			Preschool feeding		
	Average effect [average no. doses]	Average effect per 2 doses = 2*((1)/av. no. doses)	Gain per $1,000 spent = (2)*(1,000/cost of 2 treatments)^*^	Average effect [average duration in months]	Average effect per 10 mo = 10*((4)/av. duration)	Gain per $1,000 spent = (5)*(1,000/41)	Average effect [average duration in months]	Average effect per 12 mo = 12*((7)/av. duration)	Gain per $1,000 spent = (8)*(1,000/48.7)	Average effect [average duration in months]	Average effect per 12 mo = 12*((7)/av. duration)	Gain per $1,000 spent = (8)*(1,000/48.7)
	(1)	(2)	(3)	(4)	(5)	(6)	(7)	(8)	(9)	(10)	(11)	(1)
Weight	0.154	0.098	144.6	0.173	0.106	156.2	0.390	0.255	6.200	0.120	0.240	4.900
(kg)	[3.14]		[63.8, 258.7]	[3.25]		[69, 279.4]	[15.3]			[6]		
MUAC	0.198	0.113	166.5	0.198	0.113	166.5	0.310	0.135	3.300	NA	NA	NA
(cm)	[3.5]		[73.5, 258.7]	[3.5]		[73.5, 297.9]	[23]					
Height	0.087	0.054	80.0	0.095	0.056	83.0	0.380	0.248	6.100	0.270	0.540	11.100
(cm)	[3.19]		[35.3, 143.1]	[3.36]		[36.6, 148.5]	[15.3]			[6]		
Hb	0.064	0.052	76.3	0.020	0.020	30.0	−0.400	−0.174	–	0.049	0.070	1.400
(g/dL)	[2.45]		[33.7, 136.5]	[2]		[13.3, 53.7]	[23]			[8.4]		

^*^We assume a per capita cost of $0.34 for one deworming treatment. This is the current cost estimate for India (27), and it incorporates an estimate of the opportunity cost of the time that teachers spend in deworming programs, based on their wages. In square brackets, we show a lower and upper bound of the outcome gain per $1,000 spent, using the higher cost per treatment of $0.77 that GiveWell (27) estimates for African countries (also inclusive of the time of teachers) and the lower cost per treatment of $0.19 in India, if one values the opportunity cost of the time of teachers at one quarter of their wage, respectively. Estimates of the child nutrition effects of school feeding programs in LMICs come from Kristjansson et al. (23). Estimates for weight and height correspond to random effect estimates. Estimates for MUAC and hemoglobin come from a single study in Kenya referenced in ref. 23. Estimates of the child nutrition effects of preschool-feeding programs in LMICs come from Kristjansson et al. (35). Estimates for weight, height, and hemoglobin correspond to random effect estimates, no estimate of the effect on MUAC is provided in the review. $41 is the per capita cost estimate of the daily provision of a ration of 401 kcal for a 200-d school year, and $48.7 is the per capita cost estimate of the daily provision of a ration of 397 kcal for a calendar year (24).

Notes: Estimates of the average child nutrition effects of MDA correspond to our random effects estimates.

Kristjansson et al. (22, 26) conducted Cochrane Reviews on the impact of school and preschool feeding programs, respectively (22, 26). We combine their estimates of nutritional impact with information on the average duration and costs of these programs (23) to estimate the gains in nutrition outcomes per $1,000 spent in school (column 6) and preschool feeding programs (column 9).^||^ A $1,000 investment in school feeding programs results in total nutritional gains of 6.2 kg of weight, 3.3 cm of MUAC, and 6.1 cm of height. The estimated gains from MDA in settings with over 20% worm prevalence are over 23 times as large for weight, 50 times as large for MUAC, and 13 times as large for height.^**^ The relative weight and height gains of MDA are similarly large compared to preschool feeding programs. (Note that these results do not mean that school feeding is not a beneficial policy. Both school feeding and MDA may have broader benefits beyond the scope of this analysis. These results only speak to the relative cost-effectiveness of school feeding and MDA for these four nutritional outcomes.)

The cost-effectiveness of mass deworming is robust to two alternative cost estimates per person, for two doses. We consider an upper bound cost of $1.54 for African countries and a lower bound of $0.38 for India (25). These values are then used to bound estimated gains in child nutrition outcomes per $1,000 spent in deworming; these are presented in square brackets in Table 3, column 3 (and in *SI Appendix*, Table S5).

Leveraging the Bayesian interpretation of the random-effects estimator, for MDA not to be cost-effective relative to school feeding in settings with over 20% infection prevalence, we find that a policymaker would have to believe that the mean weight effect of MDA is zero with an implausible degree of precision, over 22 times as large as the posterior precision obtained with improper priors (*SI Appendix*, Table S7). The corresponding factors for MUAC and height effects are 49 and 12 times as large, respectively.

### 1.3. Comparison with Existing Meta-Analyses.

Following the release of the review by Taylor-Robinson et al. (12), we noted that one could obtain a better-powered statistical analysis by, first, including certain studies that were identified by Taylor-Robinson et al. (2015) (1) but excluded from their meta-analysis and, second, by extracting point estimates and SEs of the impact of MDA using the most precise estimators available (12). We provided a detailed discussion of these issues in a public working paper version of this study (27) and in a formal comment submitted to the Cochrane Collaboration.^††^

In Taylor-Robinson et al. (18), both the MDA and test-and-treat samples are closer to those in this paper than to those of Taylor-Robinson et al. (12, 18). However, some discrepancies remain. *SI Appendix*, Table S1 shows the differences between this paper’s sample and that of Taylor-Robinson et al. (18).^‡‡^ The robustness of our findings to differences in sample construction is examined in *SI Appendix*, Table S6.

There are also discrepancies in treatment effect estimates. For example, when examining the impact of MDA on weight, Taylor-Robinson et al. (18) do not emphasize the estimate of an increase of 0.11 kg (95% CI: −0.01, 0.24; *P* = 0.08) but a “post hoc subgroup analysis by studies published prior to and after the year 2000,” further noting that “the rationale of the cutpoint was to exclude trials carried out in the previous century when worm loads were likely to be higher.” (pp. 12–13). We argue that emphasizing such a post hoc analysis might be problematic for the following reasons. First, splitting the sample by whether a trial’s (first) article was published before or after the year 2000 is arbitrary; if one is interested in examining effects at different levels of worm load, then it makes more sense to examine this by directly accounting for worm load. Second, while there are fewer higher prevalence settings today than there were in the past, such settings still do exist, and policymakers deciding whether to implement MDA in a high-worm load setting today will find it useful to consider evidence from such settings. Third, by effectively dropping half the sample in each subgroup analysis, the power to detect a positive effect can be reduced, even if the average effect in a subgroup is larger than the overall effect.

In *SI Appendix*, section B, we examine statistical power of analyses used by Taylor-Robinson et al. and Welch et al. (12, 18, 21). *SI Appendix*, Table S2 shows none of these studies rejected the null for any of their outcomes. However, a typical meta-analysis power calculations approach (28) shows that the minimum detectable effect in previous meta-analyses was orders of magnitude larger than the minimum effect that renders deworming cost-effect relative to feeding programs. Additionally, in *SI Appendix*, section C we further discuss the meta-analysis by Welch et al. (21) and show that it is underpowered primarily because they subdivide deworming studies based on, e.g., the type of drugs used. When one relaxes these assumptions (*SI Appendix*, Table S3), one obtains statistically significant estimates of the effect of deworming on weight and in some cases, height.

**Fig. 5.**
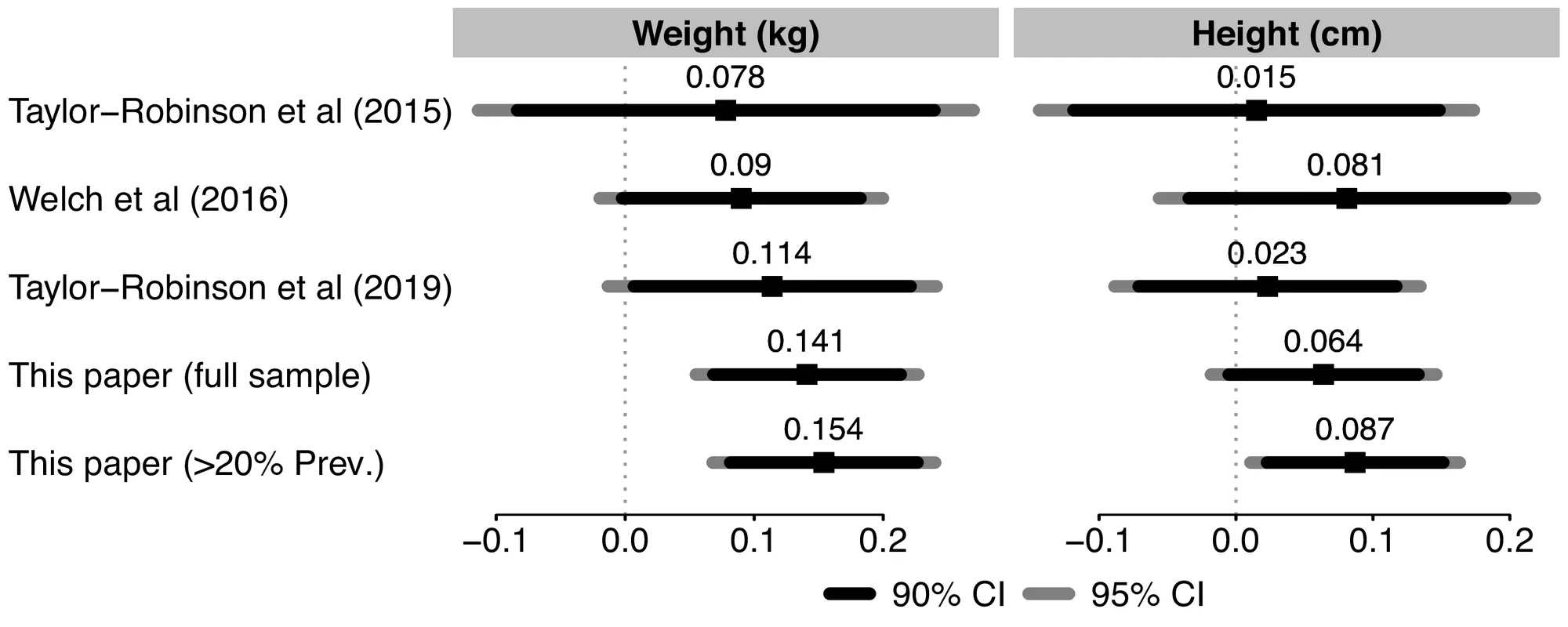
Comparison of the estimated mean impact of MDA across meta-analyses. Notes: The estimation method in Taylor-Robinson et al. (12) is random-effects for weight and fixed-effect for height. The estimation method in Welch et al. (21), Taylor-Robinson et al. (18), and this paper (for both samples) is random-effects for both outcomes. The main analysis of Welch et al. (21) is of standardized mean differences, but they present estimates of the mean effect of MDA on weight (in kg) and on height (in cm) in their “Summary of findings table” (p. 19), which we use for this graph. We back out the SEs of these estimates based on the reported CIs. The vertical dotted lines represent an effect size of zero.

In Fig. 5 we compare our estimates (and CIs) of the mean effects of MDA on weight and height to Taylor-Robinson et al. and Welch et al. (12, 18, 21). The initial estimates of the weight and height effects of MDA from Taylor-Robinson et al. (12) were small and had wide CIs (12). Welch et al. (21) and Taylor-Robinson et al. (18) incorporate additional trials but sacrifice power in other ways (e.g., splitting samples, not obtaining estimates from most precise estimators), obtaining larger point estimates with tighter CIs—but effects they estimate are still not statistically significant at the 95% level (18, 21). While we obtain point estimates that are close to those estimated by Welch et al. (21) and Taylor-Robinson et al. (18), the precision of our estimators is improved by addressing the issues above (18, 21). Consequently, we estimate statistically significant impacts, and, as expected, these are larger in settings where the WHO recommends MDA. In *SI Appendix*, Fig. S1 we conduct a more detailed analysis of whether the difference in results across different meta-analyses is driven by different inclusion/exclusion choices of studies, using more precise estimators, or using the >20% prevalence threshold instead of all studies. We find that all three of these factors contribute to differences, although their contributions vary across outcomes.

## 2. Discussion

In a meta-analysis of nutritional impacts of deworming, we find that in areas where the WHO recommends MDA (>20% prevalence), multiple-dose deworming leads to statistically significant, positive increases in child weight, MUAC, and height. We then conduct a cost effectiveness analysis and find that MDA is many times more cost-effective than widely implemented school-feeding programs. A Bayesian analysis suggests that policymakers would need extremely confident priors that MDA has no effect to not believe that it is more cost-effective than such alternative policies.

We also estimate that the implied treatment effects on infected children are higher by at least 60% than in the full sample of all trials. This gives further credence to the hypothesis that the lower average infection prevalence and intensity in MDA trials (compared to test-and-treat trials) could lead meta-analyses to be underpowered. We hope that future work may build on this finding by explicitly investigating the relationship between infection intensity and deworming effects.

The estimates in the sample are likely underestimates of the effects obtained from treating entire endemic populations because the studies in this literature generally do not address epidemiological externalities (29, 30). Most trials in the sample were randomized at the individual level, and even when trials are randomized at the cluster-level, no study, with the exception of Miguel and Kremer (30), estimates the potential epidemiological spillovers (30). Therefore, this paper’s estimates of the average effect of MDA are likely also lower bounds. The finding that deworming improves nutrition in at least some settings implies that the literature on the long-run educational and economic impacts of deworming cannot be dismissed a priori and that literature suggests the expected long-run benefits of mass deworming greatly exceed the cost (14, 16). We discuss evidence on other benefits of deworming in *SI Appendix*, section G.

Recent meta-analyses fail to reject the hypothesis that MDA has a zero-mean effect on child nutrition outcomes and argue that MDA is ineffective and should be discontinued (12, 18, 21). They advocate this policy change despite finding positive effects across several nutritional outcomes from deworming of children known to be infected. This suggests a paradox: If deworming positively affects infected individuals, one expects a smaller but positive effect from MDA in endemic populations. We show that these studies are underpowered to detect effects that would render MDA cost-effective relative to a relevant alternative policy of school-feeding. For example, by splitting their samples into different categories of analysis or by excluding relevant trials, these studies sacrifice statistical power. Our meta-analysis also has limited statistical power to detect significant effects of deworming on nutritional outcomes. Nevertheless, our analysis goes some way toward resolving the paradox by making several improvements compared to previous meta-analyses in order to obtain more precise estimates of effects.

Moving forward, policymaking can benefit from taking a decision-theoretic perspective. While the standard approach to meta-analysis focuses on whether MDA has a zero average effect, we argue that the most pressing policy question in the case of MDA is rather where MDA can be expected to be cost-effective. On the one hand, there is a consensus in the public health community that infected children should be treated and it is uncontroversial to treat very high-prevalence populations. On the other hand, there is no question that worm-free populations, or those with very low (e.g., 1%) infection prevalence, should not receive MDA. While there is uncertainty about the optimal threshold of infection prevalence or intensity that would warrant MDA, at minimum, it is evident that MDA generates nutritional gains for children in some circumstances, with larger estimated gains in settings with more infections as would be expected. This is supported by findings in this paper, which show that MDA has positive effects in settings with over 20% prevalence and is substantially more cost-effective than a leading alternative nutritional intervention.

## 3. Methods

We restrict the analysis to randomized controlled trials of MDA in which multiple doses of deworming treatment were administered and include treatment effect estimates from the longest follow-up reported. The main analysis includes trials with any of the following child nutrition indicators as outcomes: weight, MUAC, height, or hemoglobin. We focus on these outcomes because, for each, we were able to identify at least three studies examining the effects of multiple-dose deworming. We include only RCTs for which a causal intention-to-treat estimate can be obtained. Therefore, we require that the study report outcomes for the population assigned to treatment and comparison groups, independent of whether they received treatment or not.

Search procedure, data extraction, and choices of estimators are described in detail in *SI Appendix*, section A.

### 3.1. Search Procedure.

We start with the sample of studies identified by Taylor-Robinson et al. (12), for their analyses of the impact of multiple-dose deworming treatment of “all children living in an endemic area” (i.e., mass drug administration, or MDA) at longest follow-up on children’s weight, MUAC, height, and hemoglobin (12). We supplement this sample with additional studies of multiple-dose MDA identified by Welch et al. (21) that meet the trial inclusion criteria above, and we update the systematic search for MDA and “test-and-treat” trials by Taylor-Robinson et al. 2015 to identify studies published between April 14, 2015 (the Taylor-Robinson et al. search date) and June 29, 2018 (12, 21).^§§^ We also replicated Taylor Robinson et al. (12) search strategy for “test-and-treat” trials, but it has not yielded any additional studies.

### 3.2. Data Extraction and Choice of Estimator.

We follow principles detailed in the *Cochrane Handbook for Systematic Reviews of Interventions* (20), in particular deriving missing SEs from other statistics (e.g., CIs) and using the most precise estimates available (e.g., ANCOVA estimates) that are calculated based on individual-level data, if available. In addition to outcomes, we also extracted prevalence, which we defined as maximum prevalence across all worms reported in that study.

### 3.3. Meta-Analysis Models.

As deworming trials generally exhibit heterogeneity in effect sizes, likely driven by factors such as differences in infection prevalence and intensity, child age, and intervention duration, as a default we estimate a random-effects (RE) meta-analysis model. We also include fixed-effects (FE) models for comparison. Since we are particularly interested in how treatment effects may be driven by variability in worm prevalence, we estimate effects in subsets of studies with prevalence under 20%, over 20%, and over 50%. We also estimate the effect in test-and-treat trials and among all trials (MDA and test-and-treat) pooled.

In addition to the theory-agnostic approach common in public health and medicine, we also consider Bayesian estimation of the mean effect of MDA, assuming the distribution of true effects may be nonnormal and, in particular, right-skewed.^¶¶^

Next, to calculate the implied effect of deworming on infected children, we assume that effects are proportional to worm prevalence and divide the point estimate and the SE of each study by the reported prevalence. We make this assumption and then use a random-effects model to calculate the implied effect of MDA for deworming on infected children.

Last, to examine whether variation in prevalence may account for differences between MDA and test-and-treat trials, we pool data from MDA and test-and-treat trials and conduct a metaregression (with an indicator for MDA trials included).

### 3.4. Cost-Effectiveness Analysis.

We examine whether the mean nutritional benefits of MDA outweigh its costs, focusing only on the nutritional benefits of deworming. First, we search for a widely implemented intervention targeting similar outcomes in a similar population, suggesting that many policymakers consider the benefits to exceed the costs. We further require that there be a meta-analysis examining the average effect of the policy across settings and that there be data on intervention costs. Second, we compare the expected gains in child nutrition outcomes per $1,000 spent on MDA to those of this alternative intervention.

We found only one intervention meeting these criteria: preschool and school feeding programs. School feeding is implemented in over 72 countries by the World Food Programme alone (22). Kristjansson et al. (26) examine the impact of preschool feeding programs from 29 different interventions in low- and middle-income countries (26).^##^

We use a Bayesian interpretation of random-effect meta-analysis estimates to examine the degree of prior pessimism that a policymaker would need to hold about the effectiveness of MDA such that, after considering all the evidence from MDA trials, the decision-maker would be indifferent between implementing MDA and school feeding programs. To do that, we consider a policymaker with a zero-centered normal prior for $\mu_{k}$ and we define pessimism through parameter $1/v_{k}^{2}$, i.e., precision or certainty about the belief of a zero mean effect (Formally, this interpretation requires assumptions that i) a Bayesian policymaker has an uninformative or improper prior about the mean effect of MDA, ii) the cross-trial variance is known and equal to the DerSimonian and Laird (31) estimate, and iii) the true effects are normally distributed, then the posterior mean of $\mu_{k}$ corresponds to the random-effects estimate and the posterior variance of $\mu_{k}$ corresponds to the squared SE).

## Supplementary Material

Appendix 01 (PDF)

## Data Availability

Models data have been deposited in Zenodo (32).

## Footnotes

^*^Morbidity is generally agreed to depend on infection intensity (as measured by the number of worms present), rather than a simple binary infection indicator. Infection intensity, in turn, is highly skewed and strongly positively correlated with disease prevalence: severe infections are much more common in high prevalence settings (33).

^†^These calculations echo a recent epidemiological study that finds deworming to be highly cost-effective (34). In addition, multiple organizations have ranked MDA as highly cost-effective, e.g., the Copenhagen Consensus (35), the Disease Control Priorities Project (2), Givewell (25), the Abdul Latif Jameel Poverty Action Lab (36), and the World Bank (37).

^‡^Because hemoglobin and anemia have been linked to hookworm infection (3) and because only two settings in the hemoglobin sample have hookworm prevalence of over 20% (38, 39), we do not expect to detect positive effects on hemoglobin. In addition, the median length of follow-up in the sample is 1 y. Because height is typically considered to reflect a person’s cumulative nutritional status over time, longer treatment periods might be needed to detect effects on height.

^§^These samples correspond exactly to those of the analyses of children known to be infected (single dose) by Taylor-Robinson et al. (12), excluding Stephenson et al. (40), which is a misclassified MDA trial.

^¶^We do not find statistically significant differences between MDA and test-and-treat trials when including an indicator for trial type in a meta-regression. However, these comparisons are based only on 3 to 4 studies for weight, height, and MUAC, while for hemoglobin there is only one study.

^#^This cost estimate is based on data from India (41) and incorporates the cost of donated drugs, the time that teachers spend administering deworming treatment, among other costs, and thus may be somewhat higher than the costs facing a real-world policymaker.

^||^See notes for *SI Appendix*, Table S5 for details on the cost of school feeding programs.

^**^While the point estimates of the average effect of MDA on hemoglobin are positive, the point estimate of the effect of school feeding on hemoglobin is negative.

^††^We submitted this formal comment on Taylor-Robinson et al.’s review (12) to the Cochrane Collaboration on August 22, 2018, prior to Taylor-Robinson et al.’s (18) date of last search for trials (September 19, 2018). The comment can be accessed through the link: https://drive.google.com/file/d/1URJbGJfsYKDc-123U0cooskclLHo13Ic/view.

^‡‡^We do not contrast this paper’s sample to that of the Welch et al. (21) Campbell Collaboration meta-analysis since the two samples are very similar to one another (21).

^§§^Taylor-Robinson et al. (18) acknowledge that an earlier working paper version of this study (27) and the Campbell review (21) made them aware of four studies they had not included in Taylor-Robinson et al. (12) and included them in their updated review. They do not identify any additional trials which meet our trial inclusion criteria.

^¶¶^We do this for two reasons. First, deworming drugs are safe and thus are not expected to have negative effects. Second, while most MDA trials have been conducted in low prevalence and low intensity settings, several trials have been conducted in settings of moderate to high average infection intensity, which account for most of the infection morbidity. This suggests that right-skewed distributions of effects are plausible. The model we use for this is detailed in *SI Appendix*, section E.

^##^We acknowledge that school and preschool feeding programs may not be the most cost-effective nutritional interventions for these populations; however, their wide implementation suggests many policymakers consider the benefits to exceed the costs.
