# Supplementary material for: Meta-analysis and public policy: Reconciling the evidence on deworming
Source: Proc Natl Acad Sci U S A. 2024 Jun 10;121(25):e2308733121. doi: 10.1073/pnas.2308733121 (PMC11194496; doi:10.1073/pnas.2308733121)
Supplement: Supplementary file 1 — Appendix 01 (PDF) [file pnas.2308733121.sapp.pdf]

Supporting Information for

Meta-Analysis and Public Policy: Reconciling the Evidence on Deworming

Kevin Croke, Joan Hamory, Eric Hsu, Michael Kremer, Ricardo Maertens, Edward Miguel, Witold Więcek

This file includes:

- Supporting text
- Tables S1 to S8
- SI References

Supporting Information: Text

A: Details on derivation of the full analysis sample

This appendix describes how the full sample of studies included in the primary analysis was created. We first discuss the general principles we follow. Next, Section A.1 lists the studies included in our sample for which the estimates are the same as in Taylor-Robinson et al. 2015(1). Section A.2 discusses studies included in our sample which were not mentioned in Taylor-Robinson et al. 2015, and which in several cases the Taylor-Robinson et al. 2015 authors were unaware of at the time of that review (1). Section A.3 describes the process of incorporating studies that were mentioned in Taylor-Robinson et al. 2015 (1) but not included in their analyses, for example, by using formulas in *The Cochrane Handbook* (2) to derive standard errors from other reported data. The subsequent sections detail adjustments to some estimates included in the Taylor-Robinson et al. 2015 (1) sample: Section A.4 discusses cases in which more precise difference-in-differences estimators could be used instead of endline differences, while Section A.5 discusses cases in which ANCOVA estimation could be used. Section A.6 describes the process for resolving conflicting information in Awasthi and Pande (3). Section A.7 describes the correction of a data extraction error by Taylor-Robinson et al. 2015 (1) regarding the study by Awasthi et al. (4). Throughout, we discuss whether our data differ from those in the updated review by Taylor-Robinson et al. 2019 (5). Section A.8 explains how studies were classified according to WHO recommendations for MDA based on helminth prevalence. Section A.9 describes our update of Taylor-Robinson et al.'s 2015 (1) search for trials.

Inclusion/exclusion. Each paper was screened by two reviewers, although not all of them were screened fully independently. However, since almost all papers included have already been screened in other meta-analyses, we do not expect this to introduce any errors. For the extended search, two reviewers assessed inclusion/exclusion independently. There was no automation for screening.

Data extraction. Data extraction and preparation was performed by two authors, into Excel and Stata tables, following principles from Cochrane Handbook (see below). For papers included in earlier meta-analyses, extraction of values was done by one author only. The values extracted into Excel and Stata tables were also independently compared when preparing a version control repository for this project (available online) and, where needed, they were then compared with extracted values in other meta-analyses and with values in the original papers. In some cases, we reached out to authors over email to both seek clarifications and request additional data. Paper-by-paper details are described in the appendix.

Risk of bias. In assessing the risk of bias for studies included in this paper, we relied on assessments done for meta-analyses we cited. Only one paper that we include, Carmona-Fonseca and Correa-Botero (6), has not been assessed by at least one of the existing meta-analyses. We discuss its inclusion below.

For the four papers that we included, which were excluded by Taylor-Robinson et al. (Rousham and Mascie-Taylor 1994, Stoltzfus et al. 1997, Willett et al. 1979, and Wiria et al. 2013), the risk of bias was assessed by Taylor-Robinson et al. Studies by Stoltzfus et al. and Willet et al. were assessed to have selective reporting (1, 5, 7–10). Willet et al. (9) and Wiria et al. (10) were assessed to have attrition bias. Both of these categories (selective reporting and attrition bias) affected about

a quarter of studies included by Taylor-Robinson et al. 2019(5). Below we discuss why we think it is appropriate to include each of these studies on a case-by-case basis.

Study registration and protocol. We did not pre-register this study, as it was started as an update to the existing Taylor-Robinson et al. (1, 5) meta-analysis, intending to follow the Cochrane Handbook (2) guidelines and same search protocol as the original studies. Moreover, early work on this paper was done in 2015-16, before pre-registration of studies was the norm in social sciences.

Principles of data extraction and generation of results

Data extraction follows six principles derived from the *Cochrane Handbook for Systematic Reviews of Interventions* (2), which can help improve statistical power in meta-analysis and which we present below.

i. If treatment effects are presented without standard errors, standard errors are calculated using other presented data (e.g., t-statistics, p-values, or 95% confidence intervals), following the formulas provided in The Cochrane Handbook where possible (2, section 7.7.3.3).

ii. If results are reported in figures rather than in the text or in a table, Web Plot Digitizer software (11)is used to extract numerical estimates from the figures.

iii. If key information on treatment impacts is missing from a paper (and cannot be derived from what is presented), original microdata (where available) is used to obtain estimates.

iv. When possible, treatment effect estimates are extracted based on an Analysis of Covariance (ANCOVA) model. The Cochrane Handbook states that since ANCOVA estimates "give the most

precise and least biased estimates of treatment effects they should be included in the analysis when they are available" (2, section 9.4.5.2). When it is not possible to extract ANCOVA estimates, but it is possible to extract estimates based on changes from baseline to endline, this "difference-in-differences" or "changes" estimator is used. This estimator is typically more precise than the estimator based only on comparison of endline differences.

v. In case of textual contradictions about key parameter values in a trial (for example, a study text that reports significant effects versus reported test statistics that imply non-significant results), we first try to obtain the original microdata to perform the estimation ourselves. Where this is not possible, we assess which statistics were the primary focus of reporting in the text. In such cases, we also contact the original authors for clarification.

vi. Where studies report multiple treatment estimates, we follow the standard in Taylor-Robinson et al. 2015 (1) and the medical literature of favoring unadjusted estimates. If studies do not report unadjusted estimates, but they do report treatment effects adjusted with standard covariates or baseline values (such as age and sex), these estimates are included in the analysis.

Our analysis is restricted to MDA trials in which multiple doses of deworming treatment were administered and include treatment effect estimates from the longest follow-up reported. This corresponds to what Taylor-Robinson et al. 2015 (1, p. 4) term their "main comparison." In a subsequent meta-analysis, Taylor-Robinson et al. 2019 (5) broaden the main category of analysis to allow for the inclusion of multiple-dose trials that screened children for infection. However, since Taylor-Robinson et al. 2019 (5) identified no such trial, the main category of analysis remained de facto the same in the updated review. Taylor-Robinson et al. 2015 (1) identified only one multiple dose "test-and-treat" trial in each outcome category we examine, namely, Stephenson

et al. (12). This trial is, in fact, an MDA trial and is classified as such in the 2019 update. In other analyses, Taylor-Robinson et al. (1, 5) examine the effect of deworming after the first dose of treatment by combining data from multiple-dose MDA trials where effects are reported after the first dose with MDA trials of single-dose and, in the updated review, with single-dose trials that screened children for infection. We exclude single-dose MDA trials from the analysis as their length of follow-up are typically too short to allow for nutritional gains to emerge. For example, Taylor-Robinson et al. (1, 5) include Hadju et al. (13) and Palupi et al. (14) which are single-dose MDA trials with follow-up periods of 7 and 9 weeks, respectively. The median length of follow-up for multiple-dose MDA trials is one year.

A.1: Estimates adopted from Taylor-Robinson et al. (2015)

The meta-analyses by Taylor-Robinson et al. 2015 (1) of the impact of multiple dose deworming of "all children living in an endemic area'" on child weight, MUAC, height, and hemoglobin at longest follow-up include the following.

- Weight: 11 estimates from 10 studies (Alderman 2006[*] (15); Hall 2006; Sur 2005 (16); Dossa 2001a[*],b[*] (17, 18); Awasthi 2001 (3); Awasthi 2000[*] (4); Donnen 1998[*] (19); Kruger 1996a[†] (20); Watkins 1996[*] (21); Awasthi 1995/2008[*] (22, 23))
- MUAC: 4 estimates from 3 studies (Dossa 2001a[*],b[*] (17, 18); Donnen 1998[*] (19);Watkins 1996[*] (21))
- Height: 8 estimates from 7 studies (Dossa 2001a[*],b[*] (17, 18); Awasthi 2001 (3); Awasthi 2000[*] (4); Donnen 1998[*] (19); Kruger 1996a[†] (20); Watkins 1996[*] (21); Awasthi 1995/2008[*] (22, 23))

- Hemoglobin: 9 estimates from 7 studies (Ndibazza 2012 (24); Kirwan 2010 (25); Goto 2009 (26); Le Huong 2007a*,b (27); Dossa 2001a*,b* (17, 18); Awasthi 2000 (4); Kruger 1996a* (20))

Our updated sample includes 7 estimates of the weight effect, 4 of the MUAC effect, 6 of the height effect, and 5 of the effect on hemoglobin without alteration, as well as their associated standard errors. These are marked with a star in the list above. Note that the clustered, unadjusted estimates from Alderman et al. (15) of the weight effect, and the unadjusted estimates from Donnen et al. (19)of the effects on weight, height, and MUAC, were not contained in the published versions of the trials, but were obtained by Cochrane authors directly from the original trial authors. We use these same estimates in our sample. The weight and height estimates and standard errors from Kruger (20), differ slightly from what we obtain based on the published data, likely due to rounding in Taylor-Robinson et al.'s (1)data extraction procedures. These estimates, which are marked with a dagger, have been corrected in the 2019 update, and we use the latter (5).

Taylor-Robinson et al. 2019 (5) also included without alteration the estimates and standard errors marked with a star above, except for the standard error of the height effect from Dossa(17), which was revised from 0.64 to 1.099. Following the procedures from the *Cochrane* guidelines (2) for data extraction, we obtain a standard error which corresponds to the one from the 2015 review and include this one in our meta-analysis (1).

A.2: Incorporating studies not mentioned in Taylor-Robinson et al. (2015)

The full sample employed in this paper additionally incorporates five studies not mentioned in Taylor-Robinson et al. 2015(1).

Joseph et al. (28) was not included in Taylor-Robinson et al. 2015 (1) because it was published in 2015, after the final literature review was conducted for the meta-analysis. The trial targeted children between ages 1 and 2 in rural Peruvian communities over the course of 1 year. The study presents treatment effect estimates on weight and height, and their corresponding p-values, from the multiple dose treatment arm.[1] The main body of the article presents estimates using imputed child weight and height data for children lost to follow-up, but ITT estimates without any imputation can be obtained from the supplementary material (Table S4, 19), which we use in our data extraction. A formula provided in *The Cochrane Handbook* was used to compute the standard error (following Principle i). Taylor Robinson et al. 2019 (5) include this trial in their update but extract point estimates and standard errors of the weight and height effects based on data which included imputed observations (Table 4, 19). While the point estimates and standard errors for the height effect differ depending on whether imputed data were used, these are the same up to two decimal points for the weight effect.

Similarly, Carmona-Fonseca and Correa-Botero (6) was not included in Taylor-Robinson et al. 2015 (1). Carmona-Fonseca and Correa-Botero (6) is a factorial design of the provision of deworming treatment and vitamin A, targeting children under 15 years of age in a poor area of Colombia over the course of two years. The study collected baseline and endline data on child weight, height, and hemoglobin, among other outcomes. These data were generously shared with us by the study authors. We use these data to obtain ANCOVA estimates of the weight, height, and hemoglobin effects (following Principles iii and iv), based on the comparison of the deworming only and the placebo groups and the comparison of the deworming plus vitamin A and

[1] Two treatment arms involved just a single dose of deworming and were not included.

vitamin A only groups.[2] While Taylor-Robinson et al. 2019 note that they were aware of this trial while updating their review, they do not include this study in their update and note that they are "awaiting clarification on randomization" (5, p. 112). In their abstract, Carmona-Fonseca and Correa-Botero (6) state "Methodology: Clinical, randomized, controlled trial with parallel groups intervened and evaluated each 3-4 months for 4 times, followed 12 months" (p. 10) and additional details are provided in the Materials and Methods section.

Liu et al. (29) was not included in Taylor-Robinson et al. 2015 (1) because it was unavailable at the time of the review. This cluster randomized trial targeted school-aged children in China. The unadjusted difference-in-differences estimates of the treatment effect on weight, height, and hemoglobin, as well as their associated 95% confidence intervals and p-values, were obtained from the study authors, thanks to communication facilitated by the Campbell Collaboration. While p-values are reported with up to three decimal points, the bounds of the confidence intervals are presented only up to two decimal points. To calculate standard errors as precisely as possible, we use a formula provided in *The Cochrane Handbook* (2)to compute standard errors (following Principle i) based on the reported p-values. Taylor-Robinson et al. 2019 (5) also obtain data from this trial from the Campbell Collaboration. While the point estimates of the weight, height, and hemoglobin effects that they use are the same as the ones we use here, the standard errors of the weight and hemoglobin effects that they extract are larger than the ones we use. They may have calculated the standard errors from the 95% confidence intervals, the bounds of which were

[2] The deworming intervention was randomized at the household level, with all children in the same household being allocated to the same treatment group. Because data identifying siblings are not available, we are unable to cluster errors to account for within household correlation in the ANCOVA model. Positive (negative) intra-cluster correlation (ICC) would lead to under-(over-) estimation of the standard errors. However, we note that time-invariant household characteristics, which are likely to induce non-zero correlation in nutritional outcomes across siblings, are partly controlled for in the ANCOVA model due to the inclusion of the baseline dependent variable.

rounded to two decimal points. The 95% confidence intervals that they report for these outcomes do not coincide, up to two decimal points, with the ones we received from the Campbell Collaboration and which they presumably received as well.

Welch et al. (30) included two studies of multiple-dose MDA which were not identified by Taylor-Robinson et al. 2015(1): Ostwald et al. (31) and Gateff et al. (32). Ostwald et al. (31)is a trial involving school-aged children in Papua New Guinea and reports treatment effects on weight, height, and hemoglobin. Gateff et al. (32) is a study of school-aged children in rural Cameroon, and it includes weight as an outcome. Treatment effects and standard errors were calculated using information available in the published papers and formulas provided in Higgins and Green (2)(following Principle i). Taylor-Robinson et al. 2019 (5) note that these two studies were missed in the literature search conducted for Taylor-Robinson et al. 2015 (1). Taylor-Robinson et al. 2019 (5) include these two studies in their update; the estimates and standard errors that they include in their meta-analysis are the same as the ones we use here, except for the standard error of the weight effect from Gateff et al. (32). The paper reports the mean difference between treatment and control and the "standard deviation of the distribution" (*l'écart type des distributions*) (Table 3, p. 107), which Taylor-Robinson et al. 2019 (5) extract as the standard error of the treatment effect. However, it is clear from other reported statistics that this is meant to be the standard deviation of the differences in outcome between each pair of children (who had the same baseline weight), not the standard error of the treatment effect estimator. Under the interpretation of Taylor-Robinson et al. 2019 (5), the implied t-statistic would be 0.25, which differs from the paper's reported t-statistic of 2.66 and the paper's clear interpretation (in several places in the text) that the treatment effect on weight was statistically significant. If the phrase "*écart type des distributions*" is interpreted as the standard deviation of the differences in outcome between each pair, then the

implied t-statistic from the one-sample t-test is identical to the paper's reported t-statistic of 2.66. We extract this standard error by dividing the reported point estimate by this reported t-statistic.

A.3: Incorporating studies mentioned in Taylor-Robinson et al. (2015) but omitted from their meta-analyses of the effect of MDA on child weight, MUAC, height, or hemoglobin

- Willett et al. (9) is acknowledged in Taylor-Robinson et al. 2015 (1) and Taylor-Robinson et al. 2019 (5), but not included in their meta-analysis for weight gain, because the trial authors report only an adjusted treatment effect of mass deworming on weight gain and because the standard errors of the treatment effect are not directly reported in the text. Following the preference by Taylor-Robinson et al. 2015 (1) for unadjusted treatment effect estimates, we contacted the trial authors to obtain the microdata in order to extract unadjusted values, but after searching in his archives, Dr. Willett determined that the original data could no longer be located. We thus include what appears to be an adjusted treatment effect measure in our full sample (following Principle vi). The covariates used are baseline weight, study induction date (there were two separate study intakes), and age at the time of induction. All three of these are likely to improve precision of the estimators. Although treatment effect standard errors are not directly reported in the study, the reported p-value is used to calculate standard errors of treatment effects, following the procedure and formulas in Higgins and Green (2) (following Principle i). Taylor-Robinson et al. 2019 (5) do not include this study in their update because "It is unclear from the primary data whether the effect estimate is adjusted. Furthermore, it is not possible to back calculate from the ANOVA p-value to obtain the standard error for the effect estimate. It is possible to obtain the F ratio statistic from the p value and degrees of freedom (which are known),

but there are too many unknown values in the formula for the F ratio to back calculate any further.” (pp. 151-152) As noted above, the controls (potentially) included in the analysis would not lead to bias. Furthermore, we disagree with the view that the standard error cannot be calculated from the p-value. Taylor-Robinson et al. 2019 (5) acknowledge that an F ratio statistic can be calculated from the p-value which, we note, will be equivalent to the square of the t-statistic (for the two-tailed test of the hypothesis that a single coefficient is equal to zero), which can then be used to calculate the standard error using the reported point estimate (33). The Cochrane Handbook notes “Where exact P values are quoted alongside estimates of intervention effect, it is possible to estimate standard errors.” (2, p. 2:25).

- Miguel and Kremer (34) report estimated impacts on weight-for-age z-score, but do not report estimates for the raw weight outcome. As a result, this study is not included in the Taylor-Robinson et al. 2015 (1) sample for meta-analysis on weight. However, the original trial data is publicly available, and we computed the estimated impact on weight using that data and an ANCOVA specification (following data extraction principles iii and iv).[3] Schools which received treatment for schistosomiasis (praziquantel) are excluded.[4] We shared the point estimate and standard error of the weight effect we estimated with the publicly available data from Miguel and Kremer (34) with the authors of the Cochrane Review, and they incorporated these in their updated meta-analysis (5).

---

[3] Miguel and Kremer (34) correct rounding, coding, and typographical errors in the original paper and present updated data and results. We use these updated data and refer to updated results throughout, although we continue to reference Miguel and Kremer (34) for simplicity.

[4] Miguel and Kremer (34) also collected endline height and hemoglobin data, which we do not use in our updated sample. This is because there are baseline imbalances in weight and thus plausibly other nutritional indicators. While the ANCOVA strategy can account for these baseline differences for weight, we lack the data to do the same for height or hemoglobin.

- Ndibazza et al. (24) was not included in the weight or height meta-analysis by Taylor-Robinson et al. 2015 (1), likely because the study reports only impacts on related outcomes (weight-for-age, weight-for-height, and height-for-age), but not on raw weight or height. The data for this trial is not publicly available, but the Campbell Collaboration generously shared information on the raw weight and height impacts from this study obtained through correspondence with the study authors, allowing inclusion of this trial in our full sample (following Principle iii). Taylor-Robinson et al. 2019 (5) include estimates of the weight and height effect in their update, and these estimates and their standard errors correspond to the ones we use in this paper. Taylor-Robinson et al. 2019 (5) also update the standard error of the hemoglobin estimate from the 2015 review, likely based on the information provided by the Campbell Collaboration; the point estimate and standard error of the hemoglobin effect used by Taylor-Robinson et al. 2019 (5) also correspond to the ones we use in this paper.
- Wiria et al. (10) is classified in Taylor-Robinson et al. 2015 (1) as a single dose trial, but this appears to be erroneous based on our reading of the article. In their abstract, the study authors write "481 households (2022 subjects) and 473 households (1982 subjects) were assigned to receive placebo and albendazole, respectively, every three months." Furthermore, this trial reports body mass index (BMI) rather than raw weight or height outcomes in the study text. However, the Campbell Collaboration authors had contacted the original authors and received from them baseline and endline measures of weight and height, as well as standard deviations of those values for all study participants under age 16, and generously shared these estimates with us.[5] Wiria et al. (10) does not report

[5] It is not entirely clear whether the values that were calculated account for clustering, but since the household clusters had so few children per cluster, additional clustering would not substantially affect standard errors.

variance of changes, so a correlation coefficient is required to impute the standard error of the treatment effect. Correlation coefficients were estimated using a study with author-provided raw microdata of baseline and endline weight and height values (Hall et al. 2006). The estimated correlation coefficients are 0.89 for weight and 0.91 for height. Imputing these values, we estimate standard errors for Wiria et al. (10) of 0.394 and 0.535, for weight and height, respectively.[6] We thus incorporate this trial into our sample using Principles iii and iv. Taylor-Robinson et al. 2019 (5) do not include this trial in their update because "… there was a great deal of missing data. At the nine-month follow-up analysis, data were available for less than 16% of the 4004 individuals who were included in the trial (for both change score data and end values), and at the 21-month follow-up analysis, data were available for 13% of the 4004 individuals (end values data only)." (p. 18). However, this does not appear to be a correct interpretation of the study's attrition rate. While the total sample size of the trial is 4,004 participants, for multiple outcomes data was collected intentionally from only a subset of recipients. Table 2 of their paper shows that BMI was collected for only a subset of respondents (1770 out of 4004); the same table shows that 813 of these 1,770 individuals with BMI data were under 19. The Campbell Collaboration research team requested and received baseline and endline weight data for all children under 16, and they received data on 524 children under 16. Assuming equal distribution of respondents in each year from ages 0-19, the population under 16 is 84% of the population under 19, implying that the study had a sample of approximately 683 individuals under 16. The relevant attrition calculation therefore involves dividing the endline sample (524) by the number of children from whom data was collected from at baseline (683). By this

[6] Another trial for which authors provided raw microdata, Goto et al. (26), has the extremely similar baseline-endline correlation coefficients of 0.90 for weight and 0.83 for height.

calculation approximately 77% of individuals were retained to the end of the study, rather than the 13% reported by Taylor-Robinson et al. 2019 (5). This is also consistent with the article text, which reports that 80.1% of respondents were retained at endline. Thus, under the reasonable interpretation that the reason for the smaller number of weight and height observations is that data on these outcomes (like several other key outcomes in the trial) was deliberately collected from only a subset of respondents, the attrition levels in this study are not notably different than others in the sample. Therefore, we include the trial.

- Stephenson et al. 1993 (12) was included in the 2012 Cochrane Review as a case of mass treatment, and since we are examining mass treatment studies, we include this study. Prevalence at baseline was 88%, so while this is a high prevalence community, this was not a test-and-treat study, but an MDA study.[7] Departing from the previous review, Taylor-Robinson et al. 2015 (1) classify this as a study of "infected children", and do not include it in their meta-analysis of the weight, MUAC, or height effect of "all children living in an endemic area". In the 2015 update, the Cochrane Review changed its "test and treat" category, previously called "screened for infection", to "children known to be infected." Thus, in the 2015 update, Taylor-Robinson et al. 2015 (1) no longer classify Stephenson et al. 1989 (35) and Stephenson et al. 1993 (12) as mass treatment programs.[8] The distinction used in the 2012 Cochrane Review, between "test and treat" and "mass treatment",

[7] We calculate prevalence as the sample-size weighted average prevalence across all treatment and placebo arms.

[8] Taylor-Robinson et al. 2015 (1) state that "We changed the classification of Stephenson et al. (1989) and Stephenson et al. (1993). Previously these trials were in the 'all children in an endemic area' category, whereas now they are classified in the 'children with infection.' This decision was based on reviewing the trials with parasitologists and examining the prevalence and intensity of the infection where clearly the whole community was heavily infected" (1, p. 154). It is worth noting that although Taylor-Robinson et al. 2015 (1) exclude Stephenson et al. (12), they include Watkins et al. (21); the highest recorded worm baseline prevalence in Watkins et al. (21) by STH species is 91% (for ascaris); the highest prevalence in Stephenson et al. 1993 (12) is 88% (for whipworm). Thus this reclassification does not appear to have been done systematically by worm prevalence. In our view, assessing the merits of the WHO policy by including studies in environments with prevalence below WHO thresholds while excluding MDA studies in areas with high prevalence may lead to risk of bias.

corresponds more closely to the decision facing policymakers, and we preserve the original distinction. In doing so, we incorporated in our sample additional treatment effect estimates on weight, MUAC, and height from Stephenson et al. 1993 (12). These estimates measured the impact of multiple doses in an unscreened, but heavily infected, population of Kenyan schoolchildren.[9] We are able to calculate this treatment impact and standard error, following Principle i. Taylor-Robinson et al. 2019 (5) again include this study in their updated review for the analysis of multiple-dose deworming. Their point estimates and standard errors correspond to the ones we use here.

- Gupta and Urrutia (36) was excluded from the meta-analysis of the weight and height effects by Taylor-Robinson et al. 2015 (1). Regarding this study, Taylor-Robinson et al. 2015 state "[There are] only two units of allocation for relevant comparison. Children randomly divided into 4 groups, 'taking care that age distribution was similar in each group'". The 4 groups were then allocated 1 of 4 different single treatment regimens; no details given." (p. 97). We note, however, that the two-step randomization (first, of children into groups and then, of groups into experimental arms) implies that the unit of treatment allocation is the child (n=159). Following Principle i, we calculate treatment effects and standard errors from the deworming versus placebo comparisons (n=78), and the deworming plus giardia treatment versus giardia treatment only comparisons (n=81) in the published paper. Taylor-Robinson et al. 2019 (5) also include this study in their updated review for the analysis of multiple-dose deworming. Their point estimates and standard errors correspond to the ones we use here.

[9] Stephenson et al. 1989 (35) and the other treatment arm of Stephenson et al. 1993 (12) tested single dose deworming so are excluded from our analysis.

- Stoltzfus et al. (8) was excluded from the meta-analysis of the effect of MDA on weight and height in both the 2015 and 2019 Cochrane reviews; in Taylor-Robinson et al. 2019 (5) they explain that they do not include this trial in their update because it "presented data from subgroups, selected on the basis of factors such as… frequency of treatment (Stoltzfus 1997 (Cluster))… [which] were not randomized and have not been included in meta-analysis." (p. 28) The study has two treatment arms, twice- and thrice-yearly deworming, and we combine data from these two treatment arms to calculate the estimate of twice- or thrice-yearly deworming, following the procedure and formulas in Higgins and Green (2) and Principle vi. The authors present separate estimates for children under and over 10 years of age. Likewise, we calculate estimates of twice- or thrice-yearly deworming on children under and over 10 years of age, separately.
- Taylor-Robinson et al. (1, 5) include Hall 2006[10] trial in their meta-analysis of the weight effect, imputing a weight ICC coefficient from Alderman et al. (15). We use microdata, obtained from the study authors, thanks to communication facilitated by the Campbell Collaboration, to obtain ANCOVA estimates of MDA on MUAC and height (following Principles iii and iv). Taylor-Robinson et al. 2019 (5) do not include these MUAC and height estimates in their update.

  Furthermore, in a table in which Taylor-Robinson et al. 2019 (5) respond to our 2016 version of this paper (p. 154) they make the following statement about Hall et al. 2006: "It is important that this study is published, and in the public domain. The unpublished manuscript does not provide data on baseline balance. We therefore are using the change

[10] As we discuss below, we calculate treatment effects from microdata. The manuscript for this study has not been published, but is referenced in meta-analyses that make use of this study as Hall, Andrew, L Nguyen Bao Khanh, Don Bundy, N Quan Dung, T Son Hong and R Lansdown. 2006. "A randomized trial of six monthly deworming on the growth and educational achievements of Vietnamese school children."

values as provided in the manuscript and decided against the post hoc adjustment re-analysis of Croke et al. (37).The weight gain in the intervention and control group is ZERO in the unpublished manuscript; the post hoc adjusted value used in Croke et al. is 139g, SE 57G, p value 0.016." This is incorrect: in the 2016 working paper version of this paper, we report a treatment effect for Hall et al. 2006 of 0.05 kg, SE 0.06 (p. 26). We agree with Taylor-Robinson et al. on the importance of publication of this study.[11]

- Kruger et al. (20)is a factorial design study of the effect of MDA and of the provision of iron-fortified soup. While the meta-analyses of the effects on weight, height, and hemoglobin by Taylor-Robinson et al. 2015 (1) incorporate the comparison of children given deworming and unfortified soup to those given placebo and unfortified soup, they exclude the comparison of children given deworming and fortified soup to those given placebo and fortified soup. We incorporate additional estimates of the weight, height, and hemoglobin effects using the latter comparison. Kruger et al. (20) present results separately for children with low and adequate baseline iron stores. Following the procedure and formulas in Higgins and Green (2), we combine data from children with low and adequate baseline iron stores to calculate the estimated treatment effect on child weight, height, and hemoglobin, for all children.[12] Taylor-Robinson et al. 2019 (5) also include this comparison in their updated review for the analysis of multiple-dose deworming. Their point estimates and standard errors correspond to the ones we use here.

[11] As discussed in Appendix D, we also conduct a leave-each-study-out re-analysis. In the case of Hall et al. study, the effects estimated by the >20% prevalence meta-analysis model are 0.086 (compared to 0.087 with the Hall et al. study), p-value = 0.049, for height and 0.163 (compared to 0.198) for MUAC, p-value = 0.051.

[12] Taylor-Robinson et al. 2015 (1) also followed this procedure for the one comparison they included in their meta-analyses.

- Rousham and Mascie-Taylor (7) was not included in the meta-analysis of the effect of mass deworming on MUAC by Taylor-Robinson et al. 2015 (1) for reasons that are unclear to us but may be because the single usable outcome (MUAC) is presented without a standard deviation. This is a cluster-randomized study (at the village level) of the effects of deworming treatment, every 2 months, targeting children aged 2 to 6 years. Taylor-Robinson et al. 2015 (1) write "Rousham 1994 (Cluster) had 13 units of randomization (villages) containing 1476 children in total and had also not adjusted for clustering, but no outcomes from this trial were suitable for meta-analysis." (p. 16). Rousham and Mascie-Taylor (7) present difference-in-differences estimates of the effects on weight-for-age, height-for-age, and weight-for-height z-scores, as well as on MUAC, controlling for sex and age-squared. These controls are likely to increase the precision of the estimators. While the first three estimates are not suitable for inclusion in their meta-analysis of the weight or height effects, the MUAC estimate can be included in the meta-analysis of the MUAC effect. Furthermore, while TMCDG state that the analysis did not adjust for the clustered design of the trial, our interpretation is that the statistical analysis did account for clustering at the village level. Rousham and Mascie-Taylor (7) write "Since the allocation of mebendazole or placebo tablets was randomized by village rather than individual, heterogeneity of nutritional status between villages was tested using a hierarchical (nested) analysis of variance." (p. 319) We extract the difference-in-differences estimate of the MUAC effect (longest follow-up) and calculate the standard error from the reported F-statistic (following Principles i and vi). Taylor-Robinson et al. 2019 (5) do not include this study in their updated review.

Taylor-Robinson et al. (1, 5) exclude Goto et al. (26) from their meta-analysis of the effect of MDA on child weight and height but include it in their meta-analysis of the effect on hemoglobin. We received raw data generously from the study authors (via the Campbell Collaboration). However, the shared data only contained observations for children who had received the full set of intended doses of deworming medicine, rather than all who had been assigned to treatment, regardless of whether they received full treatment. Therefore, a valid intention-to-treat analysis could not be conducted and estimates from this data were not included in the meta-analyses. Taylor-Robinson 2019 (5) include this study for the analysis of the effect of multiple dose deworming on hemoglobin.

We also follow Taylor-Robinson et al. 2015 (1) and Taylor-Robinson et al. 2019 (5) in excluding Awasthi et al. (38) since the text indicates that the non-mortality outcomes such as weight were only measured for a subset of children from a randomly chosen cluster, but that *within* clusters, measured children were not chosen randomly.

A.4: Increasing precision using difference-in-differences estimators

- Sur et al. (16) is included in the meta-analysis of the weight effect by Taylor-Robinson et al. (1, 5) using an endline-only comparison. Our analysis uses additional data from the article in order to calculate a difference-in-differences estimate, following Principles ii and iv. Web Plot Digitizer software (11) was used to extract difference-in-differences estimates for Sur et al. (16) from the paper's Figure 1 (p. 265). Data from the figure and from p-values reported in the paper text were used to calculate the standard error of this estimator. The standard error of the change was calculated following the formulas and procedures in Higgins and Green (2), using information on the treatment effect, p-values, and degrees of

freedom.[13] The change in weight from baseline to endline in Sur et al. (16) is 0.2925 (note that this is a smaller treatment effect than the 0.5 difference at endline used by Taylor-Robinson et al. (1, 5)). In the text of the article Sur et al. (16) state that the p-value of this change is 0.001.[14] The t statistic is calculated using the p-value and degrees of freedom. Once the t-statistic is obtained, the standard error can be calculated using the following formula:

$$standard\ error = \frac{treatment\ effect}{t\ statistic}$$

The *tinv* function in Excel was used to determine that, given a p-value of 0.001 and a sample size of 683 (and thus 681 degrees of freedom), the t statistic is 3.3048. This, in turn, using the above formula, implies a standard error of 0.0885.[15] This revised standard error is included in our analysis. Taylor-Robinson et al. 2019 (5) acknowledge that a difference-in-differences estimate can be obtained but argue that "…in order to calculate the standard error of this effect estimate, Croke et al. (37) have assumed a t-test was used to generate the p-value for this difference in differences." The Cochrane Handbook notes "Where exact P values are quoted alongside estimates of intervention effect, it is possible to estimate standard errors." (2, p. 2:25). We follow the procedures outlined in the Cochrane handbook to extract the standard errors. We note that the assumption that a t-test was used is both consistent with the Cochrane guidelines and with Taylor-Robinson et al.'s 2019 thinking (5). In their discussion of Willet et al. (9), Taylor-Robinson et al. 2019 (5) acknowledge that an F-ratio statistic can be calculated from the p-value, and we note that this statistic is

---

[13] As the change in weight over time is not reported in the text of the paper, the same method was used that we believe Taylor-Robinson et al. 2015 (1) used to estimate the endline difference in means,

[14] See p. 261 and p. 265 of Sur et al. (16).

[15] We contacted Dr. Sur to obtain the original micro data from the trial, in order to verify these calculations directly from the original microdata. Unfortunately, Dr. Sur is now retired and thus no longer has access to the micro data.

equivalent to the square of the t statistic (for the two-tailed test of the hypothesis that a single coefficient is equal to zero) (33).

- Kirwan et al. (25) is included in the meta-analysis of the effect on hemoglobin by Taylor-Robinson et al. 2015 (1) using an endline-only comparison. Our sample uses additional data from the article in order to calculate a difference-in-differences estimate, following Principles i and iv. Taylor-Robinson et al. 2019 (5) keep the endline-only estimate in their updated review.

A.5: Increasing precision using ANCOVA

We use microdata obtained directly from the Hall et al. 2006 trial authors in order to estimate the ANCOVA specification (properly accounting for clustering) for the weight outcome, following Principles iii and iv.[16] Taylor-Robinson et al. 2019 (5) "…decided against the post hoc [ANCOVA] adjustment re-analysis of Croke et al" (p. 154). It is unclear to us whether the authors had access to the microdata which would have allowed estimation of an ANCOVA model; however, if so, Cochrane guidelines favor the use of ANCOVA estimates over difference-in-differences estimates.

[16] A second issue with this trial relates to the imputation of clustered standard errors. In Taylor-Robinson et al. 2015 (1), the treatment effect values (for a weight gain of 0.00) are included in the meta-analysis using the results reported in an unpublished manuscript obtained from the trial authors. Taylor-Robinson et al. 2015 (1) note that while some estimates were analyzed using methods to account for clustering, the main unadjusted results in the manuscript did not appear to use clustered standard errors, so they adjust the standard errors using an ICC that they obtain from Alderman et al. (15), which was a cluster randomized trial in Uganda. In this analysis the original trial data are used to calculate, rather than impute, the clustered standard errors.

A.6: Resolving apparently conflicting reporting

In the text of Awasthi and Pande (3), the authors report conflicting treatment effect estimates of the weight effect, an issue that was also noted by Taylor-Robinson et al. 2015 (1, p.43). In particular, the text of Awasthi and Pande (3) states that deworming produced positive and significant effects on weight; the authors write that "Mean (+ SE) weight gain in Kg in control versus ABZ [i.e., treatment] areas was 3.04 (0.03) versus 3.22 (0.03), (p=0.01)" (p. 823). Later in the text, however, a similar treatment effect and level of statistical significance, but a different set of standard errors for the treatment effect, is reported: "The mean weight gain in 1.5 years in the albendazole plus vitamin A group was 5.57% greater than that in the vitamin A group alone (3.22 KG (SD: 2.03, SE: 0.26) vs. 3.05 KG (SD: 1.47 SE: 0.19) P-value=0.01)." (p. 825). On one hand, the latter set of standard errors (of the treatment and control means) is consistent with the reported standard deviations, assuming that the data were averaged and analyzed at the cluster level; Taylor-Robinson et al. 2019 (5) state that this was the case, and use the latter set of standard errors. On the other hand, while the first set of standard errors is consistent with the p-value of 0.01, the set used by Taylor-Robinson et al. 2019 is not (5).[17]

We follow Principle v in consideration of this issue. Taylor-Robinson et al. 2015 (5) use the reported treatment effect for weight (0.17 kg) and appear to calculate the standard error using the second set of values (SE 0.26 and SE 0.19). Based on the p-values calculated from these numbers, and in contradiction to the p-value of 0.01 reported in the study, Taylor-Robinson et al. 2015 (1)

---

[17] While there is fundamental ambiguity in the text regarding the correct set of standard errors, we note that the one used by Taylor-Robinson et al. 2019 (5) might be upward biased and that the one we use might come from an individual-level analysis, which would be downwardly biased. Averaging data at the cluster level, as may have been done for the first set of standard errors, will over-state standard errors in the absence of perfect intra-cluster correlation. By contrast, standard errors from analysis at the individual level without appropriate clustering will understate standard errors.

refer to these results as not statistically significant, with a standard error of 0.341. By contrast the standard error is 0.0650 if one uses the p-value of 0.01 and treatment effect of 0.17 to calculate a standard error, following the formulas and procedures in Higgins and Green (2), section 7.7.3.3.[18]

Three pieces of evidence were used to assess which estimate to use. First, we consulted directly with Dr. Awasthi about this issue. She expressed disagreement with the interpretation of the results by Taylor-Robinson et al. 2015 (1), and confirmed that she agreed with the interpretation of the study's results and calculation of the study's standard errors using the p-values and effect sizes used here.[19] Second, the standard error for the weight outcome presented in the Taylor-Robinson et al. 2015 (1) analysis is 0.341, which is 1.5 to 3 times larger than the weight outcome standard errors that Taylor-Robinson et al. 2015 (1) calculate for other trials in their original sample with only a fraction of the sample size.[20] By contrast, if the standard error is calculated using the p-value and treatment effect (SE=0.0650), this makes it comparable to several other large cluster RCTs in the sample.[21] Finally, we note that it is the (statistically significant) p-value that is reported consistently in the paper, rather than the standard error. It is either the case that the authors entered incorrect measures of variance at one point in the paper, or else the authors' interpretation of the full set of study results was incorrect throughout the paper. Given our correspondence with Dr. Awasthi, the evidence from the standard errors of comparable studies, and the fact that the p-value

[18] There is yet a third possible way to calculate standard errors from data reported in this paper. This would be to use a set of standard errors reported in the abstract (0.03 for both treatment and control changes from baseline). These figures imply a still smaller standard error of 0.04.

[19] Discussion with Dr Awasthi, March 23, 2016. Dr. Awasthi was not able to share individual-level data, meaning we had to resolve this issue solely based on the values reported by the paper..

[20] For instance, Kruger et al. (20) (n=74, SE=0.2241), Watkins et al. (21) (n=226, SE=0.1059), Donnen et al. (19)(n=198, SE=0.1665), and the two treatment arms from Dossa and Ategbo (17)(n=65, SE=0.265 and n=64, SE=0.1385).

[21] For instance, Hall et al. 2006 (40 clusters, SE 0.0599), Alderman et al. (15) (50 clusters, SE=0.0892), Awasthi et al. 2008 (22) (50 clusters, SE=0.148) and several large individually randomized trials (Sur et al. (16), n=683, SE=0.0885, Awasthi et al. 2000 (4), n=1,045, SE=0.076). We do note, however, that there are two large cluster RCTs in the full sample with comparably large standard errors: Miguel and Kremer (34) (73 clusters, SE=0.44) and Wiria et al. (10) (954 household clusters, SE=0.45).

is reported consistently in the paper while the standard errors differ, the standard error derived from the p-value is incorporated into the full sample (following Principle i).

In their analysis of the height effect of mass deworming, Awasthi and Pande (3) report a single set of standard errors for mean height in the treatment and control groups; however, these are not consistent with the reported p-value of the test of the hypothesis that the effect was zero. As with weight, we derive standard errors for the treatment effect estimator based on p-values. In their 2019 update, Taylor-Robinson et al. 2019 (5) do not update the standard errors of the weight or height effects from this trial, reporting instead that they have communicated with a statistician who is familiar with the trial's analysis, and that the relationship between standard deviation and standard error in the paper's main analysis section is consistent with the trial's sample size, which they therefore believe is more accurate (p. 155).[22]

A.7: Resolving data extraction errors

The forest plots representing the meta-analysis of the hemoglobin effect in Taylor-Robinson et al. 2015 (1) switch some values for treatment and control groups in two studies: Awasthi et al. 2000 (4) and Le Huong et al. (27). The study by Awasthi et al. 2000 (4) reports the following standard deviations for hemoglobin (g/dl): 0.66g/dl (N=601) in the treatment group and 0.65g/dl (N=444) in the control group. The forest plots presented by Taylor-Robinson et al. 2015 (1) show that they extracted a s.d. of 0.65 for the treatment group and of 0.66 for the control group. They appear to

[22] They write that (p. 155) "We know that this trial was analysed by cluster (Richard Peto, pers. Com). The paper abstract and main results provides differing estimates of variance for weight gain. The abstract gives a standard error of 0.03 for weight gain in both groups, and the results gives a standard error of 0.26 in the intervention and 0.19 in the control. The data in the main results are analysed at the level of the cluster: using the relationship between SE and SD, we calculate n for the intervention as 61, and for the control 60. This corresponds (allowing for rounding errors) with the units randomised in the paper. We therefore used, for weight change, intervention 3.22 (SE 0.26) and control 3.05 (SE 0.19)."

have transposed these figures; we follow the original study's reporting of standard deviations. Similarly, Table 3 in Le Huong et al. (27) reports the following values: mean of 17.83 g/l and standard error of 0.97 g/l (N = 86) in the control group, mean of 17.54 g/l and s.e. of 0.85 g/l (N = 79) in the treatment group. The forest plots in Taylor-Robinson et al. 2015 (1) present the following extracted values: mean of 1.78 g/dl, standard deviation of 0.9 g/dl (N=86) in the treatment group, mean of 1.75 g/dl, s.d. of 0.755 g/dl (N=79) children in the control group. We follow the original study's reports of sample size, means, and standard errors.

A.8: Classification of studies by prevalence

Studies are classified according to WHO guidelines for MDA recommendations which are in turn based on whether helminth prevalence is greater than 20%, in which case MDA is recommended, and greater than 50%, in which case multiple dose MDA is recommended. Helminth prevalence in a study is classified based on the maximum prevalence across all worms reported in that study. Where possible, helminth prevalence level is classified based on prevalence described within the study itself, using cutoffs that are appropriate for WHO policy guidelines. One study in our sample is classified based on prevalence from an earlier study done in the area and which was used for targeting of the intervention, rather than baseline data collection within the trial itself (Alderman et al. (15)). Another study in our sample, Awasthi et al. 2008 (22), does not report on prevalence at all, and is classified based on two other subsequent trials conducted in the same area of India – Awasthi et al. 2000 (4) and Awasthi and Pande (3). Finally, Gateff et al. (32) is classified according to information from local health center statistics provided in the article, although the authors do not report baseline prevalence in their own sample.

For studies that screened children for infection we calculate the population-wide worm prevalence as follows.

- Yap et al. (39) report that 99.1% of children in their study area were infected with some worm species and that, among those infected, 94.3% were infected with whipworm (the most prevalent among all species). Therefore, we estimate prevalence at 0.991*0.943*100=93.4%.
- Sarkar et al. (40) report that roundworm prevalence in their study setting is 78.5%. The study does not report the prevalence of other worm species and we assume that roundworm prevalence is the highest among all species.
- Freij et al. (41) report that, among children ages 1 to 4 years in their study setting, 48.9% were infected with roundworm. The study does not report the prevalence of other worm species and we assume that roundworm prevalence is the highest among all species.
- Tee et al. (42) report that 15.3% of the children in their study population were infected with whipworm only (i.e., negative for other worm species). We contacted Dr. Lee to obtain data on worm prevalence by species (independent of co-infection), who told us that the data are no longer available. Dr. Lee also told us that he believes that whipworm is the most common infection in their setting (in Malaysia), and pointed us to the study by Huat et al. (43), in a similar setting in Malaysia, reporting that 15% of children were infected with whipworm alone, 5% with roundworm alone, and 15% with both roundworm and whipworm. We thus estimate whipworm infection prevalence in this setting as 0.153*(0.30/0.15)*100=30.6%, and assume it is the highest among all worm species.

For Awasthi 2008 (22) we impute infection prevalence using the average among all trials in our sample where prevalence is under 20%. For Gateff et al. (32) and Alderman et al. (15), we impute

infection prevalence using the average among all trials in our sample where prevalence is over 50%.

A.9: Updated search for trials

We updated the systematic search for trials by Taylor-Robinson et al. 2015 (1) to identify studies published between April 14, 2015 (the Taylor-Robinson et al. search date) and June 29, 2018.

We searched for trials in the Cochrane Central Register of Controlled Trials (CENTRAL), MEDLINE, EMBASE, and LILACS, following the search procedure outlined in the Additional Table 1, "Detailed search strategies," in Taylor-Robinson et al. (2015, pp. 127-128). The search resulted in 345 titles, of which only Carmona-Fonseca and Correa-Botero (6) and Namara et al. (44) met the inclusion criteria. Neither of these trials reported estimates of the effect of mass deworming on the child nutrition outcomes examined in this paper. Through correspondence with the respective trial authors, we received confirmation that both trials collected data on some of these outcomes. By the closure of our study update (August 10, 2018), we had received microdata from the trial by Carmona-Fonseca and Correa-Botero (6) but not from Namara et al. (44). The latter study followed up children, from a trial (24) which was discussed by Taylor-Robinson et al. 2015, for a longer period of time. While we do not include the data from the longer follow-up by Namara et al. (44), we do include the data from Ndibazza et al. (24). Further, on July 12, 2018, we looked up each study that was identified through the above methods and that met the criteria for inclusion in our study on Google Scholar. For each of these studies, we identified all the articles that cited the respective study, and that were identified by Google as meeting the following additional search criteria "albendazole OR mebendazole OR piperazine OR levamisole OR pyrantel OR tiabendazole OR thiabendazole." We added all these titles to a Google Library and listed all articles published in 2015 or later, that met the additional search criteria "weight OR

height OR MUAC OR "arm circumference" OR hemoglobin OR haemoglobin." We identified 143 titles, of which only Bhattacharyya et al. (45) met our inclusion criteria. This trial reports effects on child weight but is not included in our analysis as there are inconsistencies with the statistics that are reported (e.g., the confidence interval for the weight gain in the treatment group does not include the point estimate). We have contacted the trial authors for clarification but have not received a response (August 10, 2018).

B: Statistical power of meta-analyses in other publications

We examine whether the tests of the hypothesis that MDA has a zero average effect on child nutrition, implemented by Taylor-Robinson et al. 2015 (1), Taylor-Robinson et al. 2019 (5), and Welch et al. (30), were adequately powered to detect effects of a size that would render mass deworming cost-effective relative to feeding programs, for the outcomes analyzed in this paper.[23] Table S2 reproduces the estimates of the average child nutrition effects of MDA from the main analysis by Taylor-Robinson et al. 2015 (1), Taylor-Robinson et al. 2019 (5), and Welch et al. (30). Taylor-Robinson et al. 2015 (1) report random-effects estimates for weight, MUAC, and hemoglobin, and a fixed-effects estimate for height. Taylor-Robinson et al. 2019 (5) report random-effects estimates for weight and MUAC, and fixed-effects estimates for height and hemoglobin.[24] Welch et al. (30) report random-effects estimates in terms of standardized mean differences rather than kg (for weight) or cm (for height) so that they can combine, in a single specification, studies using different outcomes (for example, weight in kg and weight-for-age z scores); they do not report point estimates for MUAC or hemoglobin. Based on these estimates, the respective authors tested the hypothesis that MDA has a zero average effect on each outcome. As Table S2 shows, neither Taylor-Robinson et al. 2015, Taylor-Robinson et al. 2019, nor Welch et al. reject the null for any of their outcomes, at the conventional 95% confidence level (1, 5, 30). To examine whether these tests are adequately powered, we first calculate the minimum detectable effect (MDE) to reject the null hypothesis of a zero average effect at the 95%

[23] While Taylor-Robinson et al. 2019 (5) broadened the main category of analysis of their updated review, to include multiple-dose trials that screened children for infection, it remained de facto the same as they were unable to identify any such trial.

[24] For the post-hoc analyses, Taylor-Robinson et al. 2019 (5) present random-effects estimates both for the pre and post 2000 samples. For the latter analysis, the estimate of the cross-trial variance is zero and, therefore, the random-effects estimate is equivalent to the fixed-effects estimate.

confidence level, with 80% power (panel B). The minimum detectable effect for the main analysis of weight gain in Taylor-Robinson et al. 2015 (1) is 0.276 kg; this MDE is reduced in the update (5) to 0.182 kg (partly as a result of in the inclusion of some previously omitted trials), and the MDE for Welch et al. (30) is 0.294 kg.[25] We also calculate the minimum average effect that renders deworming cost-effective relative to school and preschool feeding programs (panel C).[26] The MDEs in these studies are orders of magnitude larger than the minimum effect that renders deworming cost-effect relative to feeding programs, implying that these tests lack power to reject effects that would make MDA a desirable policy option relative to other popular policies aimed at improving child nutrition in similar populations.[27] Note that Taylor-Robinson et al.'s 2019 (1) post-hoc analysis for trials published before the year 2000 has an MDE which is close to twice the size of the impact that they estimate (i.e., an increase of 0.258 kg), indicating that this analysis is also substantially underpowered.[28]

[25] For comparison, in settings with over 20% infection prevalence, our MDE is 0.122 kg for weight, 0.242 cm for MUAC, 0.108 cm for height, and 0.108 g/dl for Hb.

[26] These effects are calculated as the product of the outcome gain per dollar spent in school or preschool feeding programs and the average cost of MDA, which is calculated as the product of the cost per deworming treatment ($0.34) and the average number of doses across trials.

[27] The implicit loss function implied by requiring 95% confidence to undertake MDA without regard to the statistical power of the test is one in which there is a high cost of a false positive and a low cost of a false negative. That might be appropriate if, for example, the US Food and Drug Administration were considering a drug that might have major side effects or very high costs. However deworming drugs have already been through regulatory approval and the monetary cost of deworming is low, while there is some evidence that deworming has large long-run benefits (46). Thus, the cost of a false positive is low while the cost of a false negative is potentially substantial in endemic areas. In these situations, policymakers following the decision rule implied by a frequentist test may achieve higher welfare levels by using lower significance level thresholds, reducing the probability of incurring type II error, while incurring a greater probability of low-cost type I error (47).

[28] The weight MDE from the analysis of trials published from the year 2000 onwards (0.083 kg) is smaller than that from the analysis of trials published before the year 2000 (0.461 kg). However, even the smaller MDE is an order of magnitude larger than the effect that would render MDA cost-effective relative to school feeding (0.009 kg). That the MDE in the former analysis is smaller than in the latter is partly due to the fact that the estimated cross-trial variance in the post-2000 analysis is zero, which mechanically leads to increased precision in the estimator. However, this parameter could be biased towards zero if trials in high-prevalence settings were omitted from the analysis. We note that Taylor-Robinson et al. 2019 (5) exclude Carmona-Fonseca et al. (6) and Wiria et al. (10), both in settings with over 20% prevalence.

C: Discussion of Welch et al. (2016)

Welch et al. (30) identify many of the missing studies mentioned above, but are underpowered primarily because they subdivide deworming studies based on the type of drugs used, the frequency of treatment, and whether the trial compared deworming to pure placebo versus trials in which deworming plus an additional intervention is compared to the additional intervention alone. Thus instead of reporting a single meta-analysis which aggregates a large number of studies, they conduct multiple small sample meta-analyses.[29] In SI Table S3 we present how results change when additional studies are included. When one relaxes the narrow category of analysis from the main comparison in Welch et al. (30), for example to include trials where approved drugs aside from albendazole were used, or where deworming was done more or less frequently than twice per year, one obtains statistically significant estimates of the effect of deworming on weight, and in some cases for height.

A second reason for limited power is that Welch et al. (30)exclude two studies from their main comparison (35, 50)because they led to high heterogeneity in their meta-analyses. The authors noted that heterogeneity measured by the I^2 statistic was reduced from 93% to 61% after removing both of these studies. They scrutinized these trials and identified baseline imbalance and unclear allocation concealment, and thus decided to exclude them from their meta-analyses (p.73).

[29] For the weight effect, Welch et al. (21, p. 145) also presented "…an analysis of any mass deworming treatment (any drug, any frequency) compared to placebo, and found an effect size of 0.03 SMD for weight (95% CI: 0.00 to 0.07, 30 trials, I2=46%) with a total of 59, 691 participants. This corresponds to a difference of 0.05 kg." This analysis is not emphasized by the authors (e.g., not presented in the "Summary of findings table"). We also note that Welch et al. (30) do not exclude studies from settings with <20% infection prevalence or that administered a single dose of deworming treatment. As discussed above, treatment effect estimates from low prevalence settings are expected to be small as there are few infected children to begin with. In addition, estimates are also expected to be small in single dose trials as the length of follow-up is typically shorter than in multiple-dose trials and nutritional gains take time to occur. While we are uncertain about which trials were included in the broad-category weight analysis, in other analyses, Welch et al. (30) include Jinabhai et al. (48) and Nga et al. (49), both single dose trials with a length of follow-up of 4 months.

Stephenson et al. (35) was balanced on roundworm and whipworm prevalence and baseline height and weight, but the treatment group had higher hookworm prevalence and intensity, and the placebo group had higher ascaris intensity. The treatment group in Koroma et al. (50) had lower mean weight-for-age and height-for-age z scores, relative to the control group. However, because both studies report difference-in-differences estimates, baseline imbalances should not lead to bias unless they are correlated with changes in child weight and height. Note that the estimated weight and height effects in Koroma et al. (50) and Stephenson et al. (35) are both positive and the largest among those considered in the main analyses by Welch et al. (30).

Excluding studies because they increase measures of cross-study treatment heterogeneity will mechanically lead to downward biased estimates of cross-study variance. Further, when the distribution of true effects is right-skewed (as we discuss in the main text), excluding these estimates will lead to downward bias in estimation of average effects. When one extends Welch et al.'s (30) main comparison to include studies excluded because they increased cross-study variance, one rejects the hypothesis of zero average effect of MDA on weight (column 5, panel A) at the 99% confidence level and on height (column 5, panel B) at the 95% level. Finally, when one relaxes the category of the main analysis to include: (i) deworming trials with any drug, at any frequency, (ii) trials with vitamin A as a co-intervention, or where hygiene education was part of the treatment, and (iii) trials excluded by Welch et al. (30) because they increased cross-study heterogeneity, one rejects the null hypothesis of a zero average effect on weight and height at the 99% confidence level (column 6).

D: Robustness of random effects estimates to dropping individual study estimates and pairs of estimates (settings with >20% prevalence)

Following estimation of random-effects models, we checked whether the result for significant positive results in the settings with 20% prevalence is sensitive to exclusion of single studies or pairs of studies.

For the finding of a significant effect of MDA on child weight, MUAC, and height, one can reject the null hypothesis that the mean effect of MDA on child weight is zero at the 95% confidence level after dropping any one study estimate and after dropping any pair of estimates, among 210 possible combinations.

In these same settings, after dropping one estimate at a time, one can reject the null hypothesis of a zero-mean effect on MUAC in 4 out of 6 cases; the highest p-value is 0.056. Dropping one pair of estimates at a time, one can also reject the null for 8 pairs out of 15 possible combinations.

For height, after dropping one study estimate at a time, one can reject the null hypothesis of no average effect of MDA 94% of the time (15 out of 16), and when dropping pairs of estimates, one can reject the null 80% of the time (96 out of 120).

E: Bayesian analysis of the mean effect of MDA with skewed distributions

In this section, we turn to a Bayesian approach aimed at estimating the mean effect of MDA across settings, while allowing for skewness in the distribution of true effects. In particular, we consider the half-normal and the skew-normal distributions. While the former imposes right-skewness, the latter generalizes the normal distribution and allows for both a right and left skewness (it also allows for zero skewness). We use Stan (mc-stan.org) to estimate posterior distributions using Markov Chain Monte Carlo. All results use 12 chains with 10000 iterations for each chain (5000 used for warmup).

For the model with a half-normal distribution, we use the following prior for the scale parameter: $\tau_k \sim U(0,10)$. (Results are similar when we instead use a prior of $U(0,5)$.) For the model with a skew-normal distribution we use a prior of $U(-5,5)$ for the location parameter, of $U(0,10)$ for the scale parameter, and of $N(0, 10^2)$ for the shape or skewness parameter. (Results are similar when we instead use $U(-10,10)$ as the prior for the location parameter.)

The results are given in the SI Table S8. Panel A presents posterior mean and standard deviation for the mean effect of MDA. Both the half-normal and the skew-normal distribution produce posterior means that are similar to or slightly larger than our random-effects estimates. For instance, the posterior means for the effect size on weight gain range from 0.172 kg (skew-normal distribution) to 0.215 kg (half-normal distribution).

Panel B presents posterior distributions for the skewness parameter when using the skew-normal distribution. For weight gain, very little mass of the posterior is below zero, which supports a right-skewed distribution for effect sizes. For other outcomes, the posterior distribution looks very similar to the prior of N(0, 10), suggesting that we do not have enough data to model skewness.

F: Publication bias in the deworming literature

For each of the outcomes - height (cm), weight (kg), MUAC (cm), and hemoglobin (Hb; g/dl), we planned to test for publication bias by creating funnel plots, estimating the Egger's and Begg's tests for funnel plot asymmetry, and using the Andrews and Kasy's (51) publication bias correction technique. However, since for MUAC, we have only seven studies, less than the number recommended for tests of asymmetry of funnel plots, we do not report the test results for it.

For each outcome, we report the total number of trials included in the study, along with the number of studies that report significant effect estimates.

	Number of trials			
	$\|z\| < 1.96$	$z > 1.96$	$z < -1.96$	Total
Panel A: MDA trials only				
Weight (kg)	18	7	2	27
Height (cm)	19	2	1	22
Mid-upper arm circumference (cm)	4	2	1	7
Hb (g/dl)	12	1	0	13
Panel B: Effect on infected children, pooling MDA and test-and-treat trials				
Weight (kg)	20	9	2	31
Height (cm)	22	3	1	26
Mid-upper arm circumference (cm)	6	3	1	10
Hb (g/dl)	13	1	0	14

F.1: MDA trials only

For all three outcomes that we tested we found no evidence of funnel plot asymmetry using either Egger's or Begg's test at the conventional 5% significance level (see below).

We also used the Andrews and Kasy's (51) publication bias correction method, which provides us with the estimates of treatment effects assuming a symmetric publication bias cut-off around |z| = 1.96. This is achieved by estimating the relative probability of publication, beta_p (|z| less/more than 1.96), which in turn allows for the estimation of true mean (theta) and standard deviation across studies (hyper-SD, also often referred to in the literature as tau).

For weight, MUAC, and Hb, we cannot reject the hypothesis of relative publication probability equal to 1. However, for height, significant results are 58% more likely to be reported than insignificant results, which seems statistically significant at the 5 percent level. However, we also note that hyper-SD is 0 in the case of height and Hb, which suggests that the method used by Andrews & Kasy (51) has problems fitting data and converging on the maximum likelihood estimate.[30] In contrast, for MUAC, we only have seven studies available. Therefore, we report these results for completeness but do not put much subjective weight on them in our assessment of publication bias.

For each of the outcomes, we compare the Andrews and Kasy's (51) publication bias corrected estimates to the meta-analysis estimates. The same are presented in the table below. Overall, we

[30] This zero estimate on heterogeneity parameter could be interpreted as evidence for using the fixed effect model being more appropriate. However, anecdotally, we have observed this problem elsewhere even when heterogeneity across studies is high but when not enough studies were used with the Andrews & Kasy model of publication bias (51).

find the point estimates of effect are qualitatively unchanged.

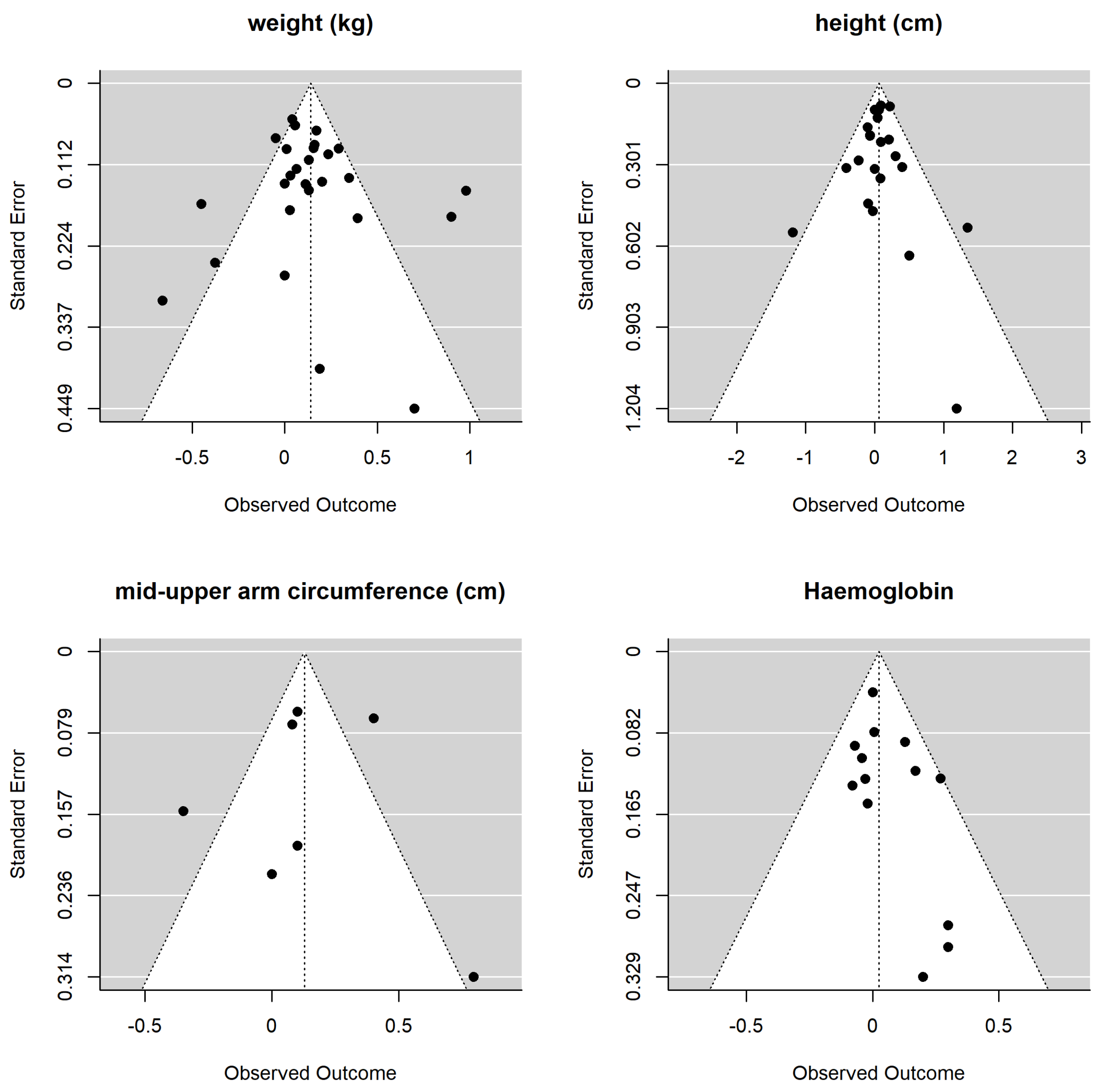

Results of publication bias assessment, MDA trials only

(A): p-values from Egger's and Begg and Mazdumar's tests

	Egger	Begg and Mazumdar
Weight (kg)	0.47	0.74
Height (cm)	0.82	0.66

Mid-upper arm circumference (cm)	0.76	0.77
Hb (g/dl)	0.14	0.37

(B) Parameters obtained from a publication bias adjustment method by Andrews and Kasy

	Theta<br>Mean (SD)	Hyper-SD<br>Mean (SD)	beta_p<br>Mean (SD)
Weight (kg)	0.18 (0.11)	0.28 (0.12)	1.95 (1.57)
Height (cm)	0.05 (0.03)	0 (0)	0.42 (0.28)
Mid-upper arm circumference (cm)	0.11 (0.12)	0.19 (0.05)	0.7 (0.94)
Hb (g/dl)	0.02 (0.02)	0 (0)	0.72 (0.77)

(C) Andrews and Kasy publication-bias corrected estimates

	Andrews-Kasy estimates<br>Mean (CI)	Meta-analysis estimates<br>Mean (CI)
Weight (kg)	0.18 (-0.03,0.39)	0.14 (0.05,0.23)
Height (cm)	0.05 (0,0.11)	0.06 (-0.02,0.15)
Mid-upper arm circumference (cm)	0.11 (-0.12,0.34)	0.13 (-0.06,0.31)
Hb (g/dl)	0.02 (-0.02,0.06)	0.03 (-0.03,0.08)

F.2: MDA and test-and-treat trials

We repeated the same tests described above for the sample of MDA and test-and-treat trials. The results for the Egger's, Begg's, and Andrews and Kasy's (51) tests are reported below.

For the test of funnel plot asymmetry, we found no evidence of funnel plot asymmetry using both the Egger's and Begg's test, for all outcomes. The probability of publishing significant results relative to insignificant results is positive for all outcomes. However, the estimates are noisy and we cannot reject the hypothesis that relative publication probability is equal to 1 (table below).

For each of the outcomes, we compared Andrews and Kasy's (51) publication bias corrected estimates to the meta-analysis estimates. The same are presented in the table below.

Results of publication bias assessment MDA trials and test-and-treat trials.

(A): p-values from Egger's and Begg and Mazdumar's tests

	Egger	Begg and Mazumdar
Weight (kg)	0.45	0.64
Height (cm)	0.87	0.69
Mid-upper arm circumference (cm)	0.42	0.38
Hb (g/dl)	0.81	1.00

(B) Parameters obtained from a publication bias adjustment method by Andrews and Kasy

	Theta<br>Mean (SD)	Hyper-SD<br>Mean (SD)	beta_p<br>Mean (SD)
Weight (kg)	0.29 (0.13)	0.39 (0.11)	2.74 (1.8)
Height (cm)	0.09 (0.06)	0.09 (0.12)	0.73 (0.8)
Mid-upper arm circumference (cm)	0.17 (0.12)	0.23 (0.04)	0.94 (0.96)
Hb (g/dl)	0.02 (0.02)	0 (0)	0.77 (0.81)

(C) Andrews and Kasy publication-bias corrected estimates

	Andrews-Kasy estimates<br>Mean (CI)	Meta-analysis estimates<br>Mean (CI)
Weight (kg)	0.29 (0.03,0.54)	0.19 (0.09,0.3)
Height (cm)	0.09 (-0.01,0.2)	0.1 (0.01,0.2)
Mid-upper arm circumference (cm)	0.17 (-0.06,0.4)	0.17 (0,0.35)
Hb (g/dl)	0.02 (-0.02,0.06)	0.02 (-0.03,0.05)

F.3: Differences in Andrews and Kasy's corrected estimates for the weight outcome

In an online appendix of their paper, Andrews and Kasy (51) use data from an earlier working version of this meta-analysis.

Their analysis is based on 22 estimates from 20 studies (compared to 27 inputs into our estimates above) and restricted to the weight outcome.

Estimating the A-K corrected estimates for the true effect using the same specification we used above (probability of publication is 1 if |z| > 1.96 and β_p if |z| < 1.96) gives us similar results to the ones reported in Andrews and Kasy (51) using the 20 studies. There are slight differences, but those may be attributed to the differences in studies used.

However, authors also comment that most studies have small but positive z-values and therefore they estimate an additional model that allows for probability of publication to change when the values of z crosses 0. That alternative model estimates that effect of MDA on weight are about ten times less likely to be published than positive ones (point estimate $\beta_{p,1}$=0.008).

This could be seen as strong evidence that estimates of deworming's impact based on published estimates are upward biased. Indeed, the bias-corrected estimates using that specification differ substantially from the ones estimated using symmetrical publication probability. In fact, the corrected estimate of the effect is then strongly negative. However, as noted by the authors, the alternative model proposed by them is a form of specification search.

F.4: Assumption of uncorrelated means and standard errors

Moreover, the method used to obtain the corrected estimate assumes that true effects of MDA are independent of the standard errors. Because the effect of MDA depends on infection intensity,

which is expected to be a non-linear function of infection prevalence (52), we can examine whether true effects are independent of standard errors by examining the correlation between prevalence and standard errors. For our full sample of weight effects, the correlation is large, at 0.342. Thus, Andrews and Kasy's (51) method for estimating selective publication probabilities may be unwarranted in the case of deworming. In general, we note that a positive correlation between true effects and standard errors will arise whenever researchers have an unbiased prior of the effect of the intervention they seek to evaluate (e.g., by conducting a pilot or by obtaining a signal) and design their interventions to obtain adequate power (e.g., the standard of 80%) to detect a significant effect at the 95% confidence level, as suggested by most guidelines on experimental design. The positive correlation arises as smaller standard errors are needed to detect smaller effects.

G: Evidence on other benefits of deworming

Ozier (53) finds that infants who lived in Kenyan communities where older school-age children were dewormed show large cognitive gains ten years later. Bleakley (54) finds that deworming campaigns in the U.S. South in the early 1900s increased school enrollment and attendance, and increased literacy and income for adults who were treated as children; Roodman (55) discusses the robustness of these results to the inclusion of additional census data. Baird et al. (56) estimate that a decade after treatment, males who participated in mass deworming in Kenya worked 17% more hours per week and had higher living standards. Females were approximately one-quarter more likely to have passed the primary-school leaving exam and attended secondary school. The estimated value of benefits, in terms of the net present value of future earnings net of increased schooling costs, exceeds the cost by more than one hundred-fold. Hamory et al. (57) study the same population in Kenya and estimate that fifteen to twenty years after treatment, treated individuals experienced a 14% gain in consumption expenditures and a 13% gain in hourly earnings, implying that the deworming intervention had an annualized social rate of return of at least 37%. Another study (37) found that a mass deworming campaign in Uganda did not have significant average educational effects among the entire population.

Supporting information: Tables

Table S1. Differences in the study inclusion and data extraction between Taylor-Robinson et al. (2019) and this paper

Paper	TMDCG inclusion or exclusion rationale	Our inclusion or exclusion rationale	TMCDG data extraction procedure	Our data extraction procedure
**Carmona-Fonseca and Correa-Botero (2015)**	**Exclude** Listed as "awaiting assessment"; "awaiting clarification on randomization"	**Include** Details for randomization are provided in the Methods section	Study not included	Obtain ANCOVA estimates using author-shared data following Principles iii and iv
**Goto et al. (2009)**	**Include**	**Exclude** Study text states that analysis was based on whether children received treatment or not, not on whether they were randomized into treatment or control (i.e. not intention-to-treat)	Calculate treatment effect based on comparison of endline values	Study not included
**Rousham and Mascie-Taylor (1994)**	**Exclude** States that analysis did not account for cluster randomization; also states that no relevant outcomes were reported.	**Include** Study text reports use of hierarchical analysis to account for clustered design; MUAC outcomes are reported.	Study not included	Calculate the standard error from the reported F-statistic and difference-in-differences estimate following Principles i and vi
**Stoltzfus et al. (1997)**	**Exclude** Treatment effects presented by subgroup rather than full sample	**Include** Treatment effect for full sample can be calculated from subgroup treatment effects	Study not included	Calculate standard errors from the reported p-value following Higgins and Green (2011) and Principle i
**Willett et al. (1979)**	**Exclude** Standard errors not presented and cannot be calculated from p-value and treatment effect; unclear whether treatment effect was adjusted for covariates	**Include** Adjusted effect can be calculated from treatment effect and p-value; include adjusted effect following Principles i and vi	Study not included	Include adjusted treatment effect following Principle vi; calculate standard error from the reported p-value following Higgins and Green (2011) and Principle i
**Wiria et al. (2013)**	**Exclude** Exclude based on large number of missing values, calculating attrition by comparing final weight/height subsample to total study population	**Include** Include because attrition should be calculated based on weight/height subsample; not on full study sample	Study not included	Estimate standard error using baseline-endline correlation coefficient from comparable studies using author-provided data following Principles iii and iv

**Sur et al. (2005)**	Included by both	Included by both	Calculate treatment effect based on comparison of end line values	Extract difference-in-differences treatment effect from graph using WebPlotDigitzer following Principle ii; calculate standard errors from reported p-values following Principle iv
**Gateff et al. (1972)**	Included by both	Included by both	Calculate treatment effect based on difference-in-differences; use reported standard deviation to calculate standard error.	Calculate treatment effect based on difference-in-differences following Principle iv; use study text reports of statistical significance to calculate standard error consistent with reported t statistic and p-value, following Principle v.
**Kirwan et al. (2010)**	Included by both	Included by both	Calculate treatment effect based on comparison of endline values	Calculate treatment effect based on difference-in-differences, following Principle iv.
**Awasthi and Pande (2001)**	Included by both	Included by both	Extract measures of variance from paper's results section.	Extract measures of variance from paper abstract; interpretation based on correspondence with author, following Principle v.
**Liu et al. (2017)**	Included by both	Included by both	Calculate treatment effect based on difference-in-differences; use 95% confidence intervals to calculate standard errors	Calculate treatment effect based on difference-in-differences; use treatment effects and p-values to calculate standard errors, following Principle i.
**Hall et al. 2006**	Included by both	Included by both	Calculate treatment effect for weight gain based on difference-in-differences as reported in manuscript; impute ICC using value from Alderman et al. (2006)	Calculate treatment effects for weight, height, and MUAC gain with ANCOVA specification using microdata provided by authors, following Principle iii.
**Dossa et al. (2001)**	Included by both	Included by both	Calculate treatment effect based on difference-in-differences	Calculate treatment effect based on difference-in-differences (calculated standard error of height differs between TMCDG and this study)
**Joseph et al. (2015)**	Included by both	Included by both	Calculate treatment effect based on difference-in-differences using sample with imputed	Calculate treatment effect based on difference-in-differences using sample without imputation

			values for missing values	

Table S2. Statistical power to detect effects that render MDA cost-effective relative to alternative policies

	Taylor-Robinson et al. (2015)				Taylor-Robinson et al. (2019)						Welch et al. (2016)	
	Weight (kg)	MUAC (cm)	Height (cm)	Hb (g/dl)	Weight (kg)	Weight (kg) *Before 2000*	Weight (kg) *Since 2000*	MUAC (cm)	Height (cm)	Hb (g/dl)	Weight (sd)	Height (sd)
	(1)	(2)	(3)	(4)	(5)	(6)	(7)	(8)	(9)	(10)	(11)	(12)
Panel A: Estimates of the child nutrition effects of MDA												
Point estimate	0.078	-0.034	0.015	-0.020	0.114	0.258	0.022	0.068	0.023	0.012	0.049	0.031
Standard error	0.098	0.109	0.081	0.031	0.065	0.164	0.029	0.130	0.057	0.031	0.034	0.027
p-val	0.426	0.757	0.852	0.524	0.079	0.116	0.449	0.603	0.681	0.698	0.150	0.255
Average doses	3.0	2.5	2.9	4.8	3.8	3.3	4.3	2.4	4.1	3.8	2.2	2
Average prevalence	45%	54%	35%	40%	45%			54%	35%	40%		
Panel B: Minimum detectable effect (in absolute units) to reject the hypothesis that the effect of MDA is zero at the 95% confidence level, with 80% power												
MDE	0.276	0.306	0.227	0.088	0.182	0.461	0.083	0.365	0.159	0.088	~0.294 kg (0.095 s.d.)	~0.195 cm (0.075 s.d.)
Panel C: Minimum average effect (in absolute units) that renders MDA cost effective relative to												
School feeding	0.006	0.003	0.006	--	0.008	0.007	0.009	0.003	0.008	--	0.005	0.004
Preschool feeding	0.005	NA	0.011	0.002	0.006	0.006	0.007	NA	0.015	0.002	0.004	0.008

Table S3. Relaxing the main category of analysis in Welch et al. (2016)

	Main Comparison	Including additional studies				
	Alb. (std.)	Other STH drugs (std.)	Alb. (other freq.)	Vitamin A; Hygiene educ.	Koroma 1996; Stephenson 1989	Other STH drugs (all freq.); Alb. (other freq.); (4); (5)
	(1)	(2)	(3)	(4)	(5)	(6)
Panel A: Weight or WAZ						
Point estimate	0.049	0.061	0.046	0.044	0.250	0.123
Standard error	0.034	0.027	0.027	0.021	0.072	0.035
p-val	0.150	0.026	0.088	0.036	0.001	0.000
N	11	14	15	14	13	28
Panel B: Height or HAZ						
Point estimate	0.031	0.026	0.044	0.054	0.180	0.094
Standard error	0.027	0.021	0.023	0.030	0.079	0.036
p-val	0.255	0.215	0.055	0.069	0.022	0.009
N	9	10	13	10	11	23

Notes: Estimation method is random-effects meta-analysis of standardized mean differences. Column 1 presents estimates of the main comparison of Welch et al. (30): albendazole twice per year (i.e., standard frequency) versus placebo. We expand the sample by including: trials with other deworming drugs at standard frequency (column 2); albendazole trials at other frequencies, when estimates for the standard frequency are not available (column 3); trials with vitamin A as a co-intervention, or where the treatment included hygiene education (column 4); trials excluded by Welch et al. (30) because they increased cross-trial heterogeneity (column 5). Column 6 expands the main comparison by considering all deworming drugs at all frequencies, as well as the additional studies from columns 4 and 5.

Table S4: Results of the meta-analysis of effects of deworming on infected children

	Weight (kg) (1)	MUAC (cm) (2)	Height (cm) (3)	Hb (g/dl) (4)
Panel A: MDA trials				
RE estimate	0.265	0.238	0.103	0.108
Standard error	0.091	0.117	0.053	0.075
p-val	0.004	0.043	0.054	0.147
N	27	7	22	13
Panel B: Test-and-treat trials				
RE estimate	0.657	0.396	0.288	-0.400
Standard error	0.336	0.167	0.154	0.434
p-val	0.050	0.018	0.061	0.356
N	4	3	4	1
Panel C: MDA and test-and-treat trials				
RE estimate	0.327	0.272	0.160	0.094
Standard error	0.096	0.099	0.062	0.074
p-val	0.001	0.006	0.010	0.203
N	31	10	26	14
Panel D: Test of the hypothesis that the average effect of deworming of infected children is the same between MDA and test-and-treat trials				
Difference	-0.407	-0.127	-0.220	0.508
Standard error	0.251	0.228	0.127	0.440
p-val	0.105	0.577	0.083	0.248

Notes: Estimation method in panels A, B, and C is random-effects. Estimation method in Panel D is random-effects meta-regression, with an indicator variable for MDA as the independent variable. Estimates are based on full sample of MDA trials. Stephenson et al. (12) is classified as an MDA trial. Point estimates and standard errors from MDA trials have been divided by infection prevalence.

Table S5. Cost-effectiveness analysis

	MDA (full sample)		
	Average effect [average no. doses]	Average effect per 2 doses = 2*((1) / av. no. doses)	Gain per $1,000 spent = (2)*(1,000 / cost of 2 treatments)†
	(1)	(2)	(3)
Weight (kg)	0.141 [3.81]	0.074	108.3 [47.8, 193.9]
MUAC (cm)	0.127 [3.57]	0.071	104.7 [46.2, 187.3]
Height (cm)	0.064 [4]	0.032	47.3 [20.9, 84.7]
Hb (g/dl)	0.026 [3.92]	0.013	19.2 [8.5, 34.3]

Notes:

Estimates of the average child nutrition effects of MDA correspond to our random effects estimates. † We assume a per capita cost of $0.34 for one deworming treatment. This is the current cost estimate for India (58), and it incorporates an estimate of the opportunity cost of the time that teachers spend in deworming programs, based on their wages. In square brackets we show a lower and upper bound of the outcome gain per $1,000 spent, using the higher cost per treatment of $0.77 that GiveWell (58) estimates for African countries (also inclusive of the time of teachers) and the lower cost per treatment of $0.19 in India, if one values the opportunity cost of the time of teachers at one quarter of their wage, respectively. (Muralidharan and Sundararaman (59) show that public school teachers in India obtain large rents, with average wages being over four times as large as those of private sector teachers.)

We use the estimate from Kristjansson et al. 2016 (60) of the average per capita cost of providing school-based food supplementation for a school year of 200 days (10 months) and for a fixed daily ration of 401 kcal (60), specifically, the average caloric content in the school feeding programs included in an unpublished update of the Kristjansson et al. 2007 (61) review; this average cost is $41. Following Kristjansson et al. 2016 (60), and if the cost per calorie delivered in school and preschool feeding programs are the same, we estimate the per capita cost of a one calendar year provision of a daily ration of 391 kcal, the average in the preschool feeding programs reviewed by Kristjansson et al. 2015 (62) at $41*1.2*(397/401) = $48.70.

Table S6. Robustness to exclusion of additional studies

	Weight	MUAC	Height	Hb
	(1)	(2)	(3)	(4)
Panel A: MDA trials with ≥20% prevalence				
Point estimate	0.154	0.198	0.087	0.064
Standard error	0.044	0.086	0.039	0.038
p-val	<0.001	0.022	0.024	0.098
N	21	6	16	11
Panel B: Robustness Analysis				
B1. To dropping studies not mentioned by Taylor-Robinson et al. (2015)				
Point estimate	0.146	0.198	0.095	0.086
Standard error	0.053	0.086	0.041	0.057
p-val	0.005	0.022	0.021	0.133
N	16	6	12	7
B2. To dropping studies erroneously not classified as MDA by Taylor-Robinson et al. (2015)				
Point estimate	0.131	0.108	0.091	0.064
Standard error	0.035	0.056	0.040	0.038
p-val	<0.001	0.056	0.022	0.098
N	19	5	14	11
B3. To dropping studies not included in Taylor-Robinson et al. (2015) likely because standard errors are not directly reported or because only adjusted estimates are available				
Point estimate	0.149	0.198	0.066	0.064
Standard error	0.053	0.086	0.048	0.038
p-val	0.005	0.022	0.166	0.098
N	18	6	14	11
B4. To dropping studies not included in Taylor-Robinson et al. (2015) likely because only estimates on "related" measures were presented (e.g. weight-for-age Z score instead of weight)				
Point estimate	0.168	0.198	0.087	0.064
Standard error	0.041	0.086	0.039	0.038
p-val	<0.001	0.022	0.024	0.098
N	20	6	16	11

Notes: Panel B.1 excludes Joseph et al. (28), Carmona-Fonseca and Correa-Botero (6), Liu et al. (29), Ostwald et al. (31) and Gateff et al. (32). Panel B.2 excludes Wiria et al. (10) and Stephenson et al. (12) . Panel B.3 excludes Willett et al. (9) and Stoltzfus et al. (8). Panel B.4 excludes Miguel and Kremer (34) and Ndibazza et al. (24).

Table S7. Pessimism of Priors

	MDA (>20% prevalence)			School feeding			Preschool feeding		
	Gain per $1,000 spent†	Standar d error of RE estimate	Posterior precision with improper prior = (1)/(2)^2	Gain per $1,000 spent	Minimum pessimism (MDA) that leads to indifference = ((1)/(4) - 1)*(3)	Minimum relative pessimism (MDA) that leads to indifference‡ = ((1)/(4) - (1))	Gain per $1,000 spent	Minimum pessimism (MDA) that leads to indifference = ((1)/(7) - (1))*(3)	Minimum relative pessimism (MDA) that leads to indifference‡ = ((1)/(7) - (1))
	(1)	(2)	(3)	(4)	(5)	(6)	(7)	(8)	(9)
Weight (kg)	144.6	0.111	80.8	6.2	1797.9	22.3	4.9	2289.7	28.3
MUAC (cm)	166.5	0.154	42.1	3.3	2092.0	49.6	NA	NA	NA
Height (cm)	80.0	0.181	30.6	6.1	374.0	12.2	11.1	190.5	6.2
Hb (g/dl)	76.3	0.038	706.2	--	--	--	1.4	36768.1	52.1

Notes: Estimates of the average child nutrition effects of MDA correspond to our random effects estimates. † We assume a per capita cost of $0.34 for one deworming treatment. This is the current cost estimate for India (58), and it incorporates an estimate of the opportunity cost of the time that teachers spend in deworming programs, based on their wages. Estimates of the child nutrition effects of school feeding programs in low and middle income countries come from the systematic review by Kristjansson et al. 2007 (61). Estimates for weight and height correspond to random effect estimates. Estimates for MUAC and hemoglobin come from a single study in Kenya (61). Estimates of the child nutrition effects of preschool-feeding programs in low and middle income countries come from the systematic review by Kristjansson et al. (62). Estimates for weight, height, and hemoglobin correspond to random effect estimates, no estimate of the effect on MUAC is provided in the review. $41 is the per capita cost estimate of the daily provision of a ration of 401kcal for a 200-day school year, and $48.7 is the per capita cost estimate of the daily provision of a ration of 397 kcal for a calendar year (60). ‡ Measures the minimum level of pessimism about the mean effect of MDA that would lead to indifference with feeding programs, relative to the posterior precision of the mean MDA effect that obtains with an improper prior.

Table S8. Bayesian meta-analysis model of MDA trials using skewed distributions of mean effect

	Weight (kg)	MUAC (cm)	Height (cm)	Hb (g/dl)
Panel A: Posterior mean effect across settings (s.d.)				
Half-normal	0.215	0.231	0.069	0.046
	(0.043)	(0.11)	(0.037)	(0.034)
Skew-normal	0.172	0.137	0.064	0.037
	(0.057)	(0.188)	(0.045)	(0.04)
Panel B: Posterior mean for skewness parameter (s.d.)				
Skew-normal	9.046	0.105	-0.501	0.018
	(5.778)	(10.012)	(10.414)	(10.375)

Notes: For models in which the true average MDA effect is drawn from a half-normal distribution, we used a uniform prior for the parameter with support from zero to ten. For models in which the true average effect of MDA deworming is drawn from a skew-normal distribution, we used uniform priors for the location and scale parameters with support from -5 to 5 and 0 to 10, respectively. For the shape parameter we used a normal prior centered at zero with variance 100.

Supporting information: Figures

Figure S1. Random effects estimates under different combinations of studies sample and data extraction procedures

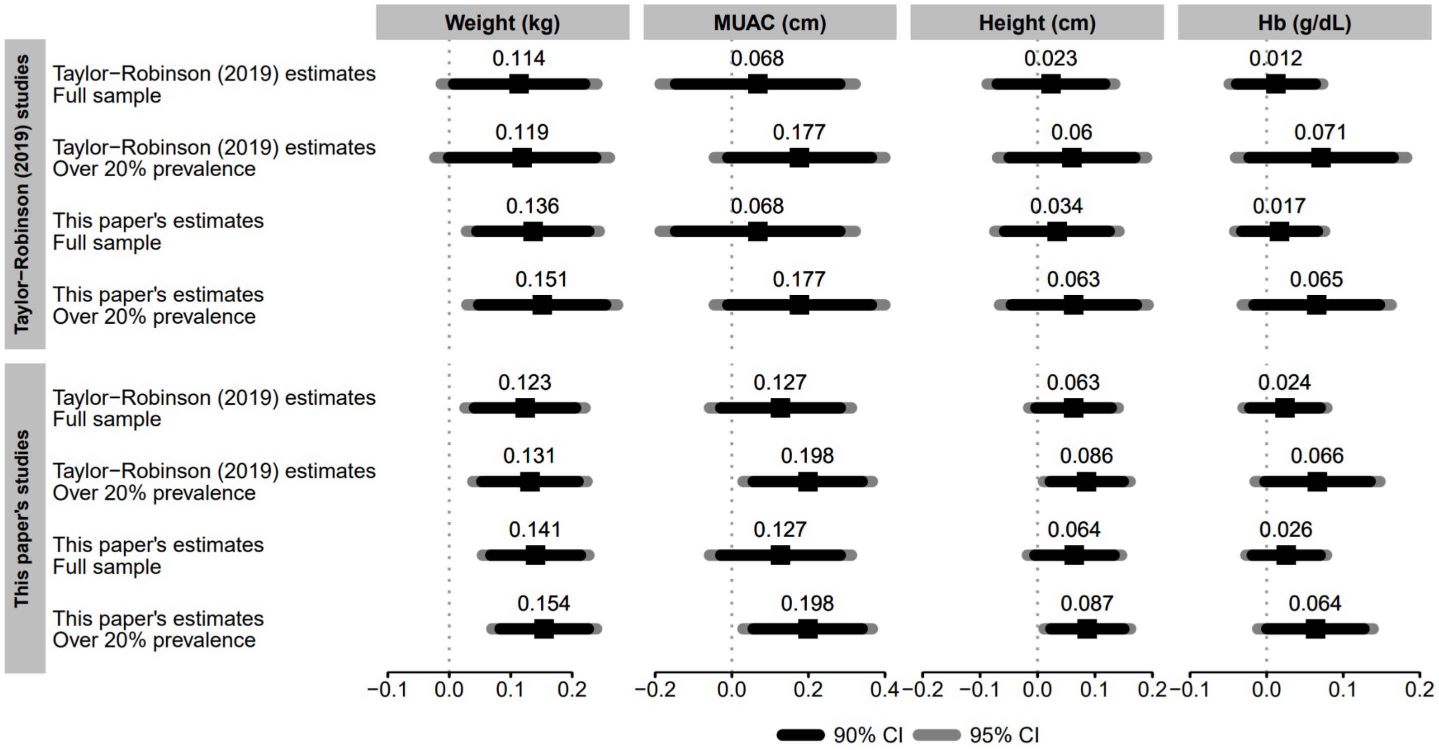

Notes: estimates using this paper's data and sample are the same as those presented in Table 2. The full sample of studies in Taylor-Robinson et al. 2019 (5) includes 21 estimates of effects on weight (from 18 different studies), 5 estimates of effects on MUAC (from 4 different studies), 16 estimates of effects on height (from 13 different studies), and 12 estimates of effects on hemoblogin (from 9 different studies). We did not extract data from Goto (26), and therefore estimates in this figure that use Taylor-Robinson et al. 2019 (5) studies and this paper's data use one less observation than those using data from Taylor-Robinson et al. 2019 (5). The sample of studies in Taylor-Robinson et al. 2019 (5) with a prevalence of 20% or higher includes 15 estimates of effects on weight, 4 estimates of effects on MUAC, 10 estimates of effects on height, and 9 estimates of effects on hemoblogin.

Supporting Information: References

1. D. C. Taylor-Robinson, N. Maayan, K. Soares-Weiser, S. Donegan, P. Garner, Deworming drugs for soil-transmitted intestinal worms in children: effects on nutritional indicators, haemoglobin, and school performance. *Cochrane Database Syst. Rev.* **2015**, CD000371, (2015).
2. J. P. T. Higgins, S. Green, *Cochrane Handbook for Systematic Reviews of Interventions* (Cochrane, 2011).
3. S. Awasthi, V. K. Pande, Six-monthly de-worming in infants to study effects on growth. *Indian J. Pediatr.* **68**, 823–827, (2001).
4. S. Awasthi, V. K. Pande, R. H. Fletcher, Periodic deworming with albendazole and its impact on growth status and diarrhoeal incidence among children in an urban slum of India. *Indian Pediatr.* **37**, 19–29, (2000).
5. D. C. Taylor-Robinson, N. Maayan, S. Donegan, M. Chaplin, P. Garner, Public health deworming programmes for soil-transmitted helminths in children living in endemic areas. *Cochrane Database Syst. Rev.* **9**, CD000371, (2019).
6. J. Carmona-Fonseca, A. Correa-Botero, Efecto del albendazol y la Vitamina A periódicos sobre helmintos intestinales y anemia en niños del Urabá antioqueño (Colombia). *Biosalud* **14**, 9–25, (2015).
7. E. K. Rousham, C. G. Mascie-Taylor, An 18-month study of the effect of periodic anthelminthic treatment on the growth and nutritional status of pre-school children in Bangladesh. *Ann. Hum. Biol.* **21**, 315–324, (1994).
8. R. J. Stoltzfus, M. Albonico, J. M. Tielsch, H. M. Chwaya, L. Savioli, School-based deworming program yields small improvement in growth of Zanzibari school children after one year. *J. Nutr.* **127**, 2187–2193, (1997).
9. W. C. Willett, W. L. Kilama, C. M. Kihamia, Ascaris and growth rates: a randomized trial of treatment. *Am. J. Public Health* **69**, 987–991, (1979).
10. A. E. Wiria, *et al.*, The Effect of Three-Monthly Albendazole Treatment on Malarial Parasitemia and Allergy: A Household-Based Cluster-Randomized, Double-Blind, Placebo-Controlled Trial. *PLOS ONE* **8**, 1–9, (2013).
11. A. Rohatgi, WebPlotDigitizer, (2015), . Deposited 2015.
12. L. S. Stephenson, M. C. Latham, E. J. Adams, S. N. Kinoti, A. Pertet, Physical fitness, growth and appetite of Kenyan school boys with hookworm, Trichuris trichiura and Ascaris lumbricoides infections are improved four months after a single dose of albendazole. *J. Nutr.* **123**, 1036–1046, (1993).
13. V. Hadju, *et al.*, Improvements in appetite and growth in helminth-infected schoolboys three and seven weeks after a single dose of pyrantel pamoate. *Parasitology* **113 (Pt 5)**, 497–504, (1996).
14. L. Palupi, W. Schultink, E. Achadi, R. Gross, Effective community intervention to improve hemoglobin status in preschoolers receiving once-weekly iron supplementation. *Am. J. Clin. Nutr.* **65**, 1057–1061, (1997).
15. H. Alderman, J. Konde-Lule, I. Sebuliba, D. Bundy, A. Hall, Effect on weight gain of routinely giving albendazole to preschool children during child health days in Uganda: cluster randomised controlled trial. *BMJ* **333**, 122–124, (2006).
16. D. Sur, D. R. Saha, B. Manna, K. Rajendran, S. K. Bhattacharya, Periodic deworming with

albendazole and its impact on growth status and diarrhoeal incidence among children in an urban slum of India. *Trans. R. Soc. Trop. Med. Hyg.* **99**, 261–267, (2005).
17. R. A. Dossa, E. A. Ategbo, F. L. de Koning, J. M. van Raaij, J. G. Hautvast, Impact of iron supplementation and deworming on growth performance in preschool Beninese children. *Eur. J. Clin. Nutr.* **55**, 223–228, (2001).
18. R. A. Dossa, E. A. Ategbo, J. M. Van Raaij, C. de Graaf, J. G. Hautvast, Multivitamin-multimineral and iron supplementation did not improve appetite of young stunted and anemic Beninese children. *J. Nutr.* **131**, 2874–2879, (2001).
19. P. Donnen, *et al.*, Vitamin A supplementation but not deworming improves growth of malnourished preschool children in eastern Zaire. *J. Nutr.* **128**, 1320–1327, (1998).
20. M. Kruger, C. J. Badenhorst, E. P. G. Mansvelt, J. A. Laubscher, A. J. S. Benadé, Effects of Iron Fortification in a School Feeding Scheme and Anthelmintic Therapy on the Iron Status and Growth of Six- to Eight-Year-Old Schoolchildren. *Food Nutr. Bull.* **17**, 1–11, (1996).
21. W. E. Watkins, J. R. Cruz, E. Pollitt, The effects of deworming on indicators of school performance in Guatemala. *Trans. R. Soc. Trop. Med. Hyg.* **90**, 156–161, (1996).
22. S. Awasthi, *et al.*, Effects of deworming on malnourished preschool children in India: an open-labelled, cluster-randomized trial. *PLoS Negl. Trop. Dis.* **2**, e223, (2008).
23. S. Awasthi, R. Peto, R. Fletcher, H. Glick, Treating Parasitic Infestations in Children [Monograph No. 3], (1995).
24. J. Ndibazza, *et al.*, Impact of Anthelminthic Treatment in Pregnancy and Childhood on Immunisations, Infections and Eczema in Childhood: A Randomised Controlled Trial. *PLOS ONE* **7**, 1–14, (2012).
25. P. Kirwan, *et al.*, Impact of repeated four-monthly anthelmintic treatment on Plasmodium infection in preschool children: a double-blind placebo-controlled randomized trial. *BMC Infect. Dis.* **10**, 277, (2010).
26. R. Goto, C. G. N. Mascie-Taylor, P. G. Lunn, Impact of anti-Giardia and anthelminthic treatment on infant growth and intestinal permeability in rural Bangladesh: a randomised double-blind controlled study. *Trans. R. Soc. Trop. Med. Hyg.* **103**, 520–529, (2009).
27. T. Le Huong, I. D. Brouwer, K. C. Nguyen, J. Burema, F. J. Kok, The effect of iron fortification and de-worming on anaemia and iron status of Vietnamese schoolchildren. *Br. J. Nutr.* **97**, 955–962, (2007).
28. S. A. Joseph, *et al.*, The Effect of Deworming on Growth in One-Year-Old Children Living in a Soil-Transmitted Helminth-Endemic Area of Peru: A Randomized Controlled Trial. *PLoS Negl. Trop. Dis.* **9**, 1–20, (2015).
29. C. Liu, *et al.*, Effect of Deworming on Indices of Health, Cognition, and Education Among Schoolchildren in Rural China: A Cluster-Randomized Controlled Trial. *Am. J. Trop. Med. Hyg.* **96**, 1478–1489, (2017).
30. V. A. Welch, *et al.*, Deworming and adjuvant interventions for improving the developmental health and well-being of children in low- and middle-income countries: a systematic review and network meta-analysis. *Campbell Syst. Rev.* **12**, 1–383, (2016).
31. R. Ostwald, *et al.*, The effect of intestinal parasites on nutritional status in well-nourished school age children in Papua New Guinea. *Nutr. Rep. Int.* **30**, 1409–1421, (1984).
32. C. Gateff, G. Lemarinier, R. Labusquiere, Chimiotherapie antihelminthique systematique au thiabendazole en milieu scolaire africain. *Ann. Société Belge Médecine Trop.* **52**, 103–112, (1972).
33. J. Stock, M. Watson, *Introduction to Econometrics (3rd edition)* (Addison Wesley

Longman, 2011).
34. E. Miguel, M. Kremer, Worms: Identifying Impacts on Education and Health in the Presence of Treatment Externalities. *Econometrica* **72**, 159–217, (2004).
35. L. S. Stephenson, M. C. Latham, K. M. Kurz, S. N. Kinoti, H. Brigham, Treatment with a single dose of albendazole improves growth of Kenyan schoolchildren with hookworm, Trichuris trichiura, and Ascaris lumbricoides infections. *Am. J. Trop. Med. Hyg.* **41**, 78–87, (1989).
36. M. C. Gupta, J. J. Urrutia, Effect of periodic antiascaris and antigiardia treatment on nutritional status of preschool children. *Am. J. Clin. Nutr.* **36**, 79–86, (1982).
37. K. Croke, R. Atun, The long run impact of early childhood deworming on numeracy and literacy: Evidence from Uganda. *PLoS Negl. Trop. Dis.* **13**, e0007085, (2019).
38. S. Awasthi, *et al.*, Population deworming every 6 months with albendazole in 1 million pre-school children in North India: DEVTA, a cluster-randomised trial. *Lancet Lond. Engl.* **381**, 1478–1486, (2013).
39. P. Yap, *et al.*, Effect of deworming on physical fitness of school-aged children in Yunnan, China: a double-blind, randomized, placebo-controlled trial. *PLoS Negl. Trop. Dis.* **8**, e2983, (2014).
40. N. R. Sarkar, K. S. Anwar, K. B. Biswas, M. A. Mannan, Effect of deworming on nutritional status of ascaris infested slum children of Dhaka, Bangladesh. *Indian Pediatr.* **39**, 1021–1026, (2002).
41. L. Freij, G. W. Meeuwisse, N. O. Berg, S. Wall, M. Gebre-Medhin, Ascariasis and malnutrition. A study in urban Ethiopian children. *Am. J. Clin. Nutr.* **32**, 1545–1553, (1979).
42. M. H. Tee, Y. Y. Lee, N. A. Majid, N. M. Noori, S. M. Raj, Growth reduction among primary schoolchildren with light trichuriasis in Malaysia treated with albendazole. *Southeast Asian J. Trop. Med. Public Health* **44**, 19–24, (2013).
43. L. B. Huat, *et al.*, Prevalence and risk factors of intestinal helminth infection among rural malay children. *J. Glob. Infect. Dis.* **4**, 10–14, (2012).
44. B. Namara, *et al.*, Effects of treating helminths during pregnancy and early childhood on risk of allergy-related outcomes: Follow-up of a randomized controlled trial. *Pediatr. Allergy Immunol. Off. Publ. Eur. Soc. Pediatr. Allergy Immunol.* **28**, 784–792, (2017).
45. M. Bhattacharyya, R. N. Sinha, A. Sarkar, A. K. Mallick, A. K. Panda, Effect on weight after Albendazole therapy among the primary school children in a slum of Kolkata. *Natl. J. Community Med.* **9**, 106–109, (2018).
46. A. Ahuja, *et al.*, When Should Governments Subsidize Health? The Case of Mass Deworming. *World Bank Econ. Rev.* **29**, S9–S24, (2015).
47. C. Manski, Partial Identification of Counterfactual Choice Probabilities. *Int. Econ. Rev.* **48**, 1393–1410, (2007).
48. C. C. Jinabhai, *et al.*, Epidemiology of helminth infections: implications for parasite control programmes, a South African perspective. *Public Health Nutr.* **4**, 1211–1219, (2001).
49. T. T. Nga, *et al.*, Multi-micronutrient-fortified biscuits decreased prevalence of anemia and improved micronutrient status and effectiveness of deworming in rural Vietnamese school children. *J. Nutr.* **139**, 1013–1021, (2009).
50. M. M. Koroma, R. A. Williams, R. de la Haye R, M. Hodges, Effects of albendazole on growth of primary school children and the prevalence and intensity of soil-transmitted helminths in Sierra Leone. *J. Trop. Pediatr.* **42**, 371–372, (1996).

51. I. Andrews, M. Kasy, Identification of and Correction for Publication Bias. *Am. Econ. Rev.* **109**, 2766–94, (2019).
52. R. M. Anderson, R. M. May, "Helminth Infections of Humans: Mathematical Models, Population Dynamics, and Control" in Advances in Parasitology., J. R. Baker, R. Muller, Eds. (Academic Press, 1985), , pp. 1–101.
53. O. Ozier, Exploiting Externalities to Estimate the Long-Term Effects of Early Childhood Deworming. *Am. Econ. J. Appl. Econ.* **10**, 235–62, (2018).
54. H. Bleakley, Disease and Development: Evidence from Hookworm Eradication in the American South. *Q. J. Econ.* **122**, 73–117, (2007).
55. D. Roodman, Comment: The impacts of the hookworm eradication in the American South, (2017).
56. S. Baird, J. H. Hicks, M. Kremer, E. Miguel, Worms at Work: Long-run Impacts of a Child Health Investment. *Q. J. Econ.* **131**, 1637–1680, (2016).
57. J. Hamory, E. Miguel, M. Walker, M. Kremer, S. Baird, Twenty-year economic impacts of deworming. *Proc. Natl. Acad. Sci.* **118**, e2023185118, (2021).
58. GiveWell, "Evidence Action's Deworm the World Initiative" (GiveWell, 2017).
59. K. Muralidharan, V. Sundararaman, The Aggregate Effect of School Choice: Evidence from a Two-Stage Experiment in India *. *Q. J. Econ.* **130**, 1011–1066, (2015).
60. E. A. Kristjansson, *et al.*, Costs, and cost-outcome of school feeding programmes and feeding programmes for young children. Evidence and recommendations. *Int. J. Educ. Dev.* **48**, 79–83, (2016).
61. E. A. Kristjansson, *et al.*, School feeding for improving the physical and psychosocial health of disadvantaged elementary school children. *Cochrane Database Syst. Rev.*, CD004676, (2007), https:/doi.org/10.1002/14651858.CD004676.pub2.
62. E. Kristjansson, *et al.*, Food supplementation for improving the physical and psychosocial health of socio-economically disadvantaged children aged three months to five years. *Cochrane Database Syst. Rev.* **2015**, CD009924, (2015).
